# Extraoral Taste Receptor Discovery: New Light on Ayurvedic Pharmacology

**DOI:** 10.1155/2017/5435831

**Published:** 2017-05-31

**Authors:** Marilena Gilca, Dorin Dragos

**Affiliations:** ^1^Biochemistry Department, Faculty of General Medicine, “Carol Davila” University of Medicine and Pharmacy, B-dul “Eroilor Sanitari” No. 8, Sector 6, 76241 Bucharest, Romania; ^2^Medical Semiology Department, Faculty of General Medicine, “Carol Davila” University of Medicine and Pharmacy, B-dul “Eroilor Sanitari” No. 8, Sector 6, 76241 Bucharest, Romania; ^3^Nephrology Clinic, University Emergency Hospital Bucharest, Bucharest, Romania

## Abstract

More and more research studies are revealing unexpectedly important roles of taste for health and pathogenesis of various diseases. Only recently it has been shown that taste receptors have many extraoral locations (e.g., stomach, intestines, liver, pancreas, respiratory system, heart, brain, kidney, urinary bladder, pancreas, adipose tissue, testis, and ovary), being part of a large* diffuse chemosensory system*. The functional implications of these taste receptors widely dispersed in various organs or tissues shed a new light on several concepts used in ayurvedic pharmacology (*dravyaguna vijnana*), such as taste (*rasa*), postdigestive effect (*vipaka*), qualities (*guna*), and energetic nature (*virya*). This review summarizes the significance of extraoral taste receptors and transient receptor potential (TRP) channels for ayurvedic pharmacology, as well as the biological activities of various types of phytochemical tastants from an ayurvedic perspective. The relative importance of taste (*rasa*), postdigestive effect (*vipaka*), and energetic nature (*virya*) as ethnopharmacological descriptors within Ayurveda boundaries will also be discussed.

## 1. Introduction

Until recently, the essential role of taste was considered to be the detection of nutritious and poisonous substances. Accumulating evidence indicates that taste receptors mediate diverse important nontasting roles through various specialized mechanisms. This perspective is closer to Ayurveda vision on taste (Sanskrit* rasa*).

Concerning the number of taste modalities, modern science recognized five (sweet, bitter, salty, sour, and umami), while Ayurveda six (*madhura*: sweet,* lavana*: salty,* amla*: sour,* katu*: pungent,* tikta*: bitter, and* kashaya*: astringent).

The sense of taste is governed by distinct cell types located in the taste buds that express only one type of specific taste receptor (TR). There are four categories of taste bud cells: type I, type II, type III, and type IV. Type II cells (or receptor cells) are involved in bitter, sweet, and umami detection, while type III (or presynaptic cells) in sour taste perception [1–4]. It is not yet clear whether type I (glial-like supporting cells similar to astrocytes) [5] or type III cells (presynaptic cells) play the main role in salty taste perception [6–8]. Type IV cells are basal cell type, responsible for renewal of taste bud cells and mechanoreception [9, 10].

Sweet, bitter, umami tastes, and probably astringency trigeminal orosensation also, are mediated via G protein-coupled receptors, while salty and sour tastes, as well as pungency trigeminal orosensation, involve different systems, which include specialized membrane ion channels [1, 2, 11–13].

Interestingly, since their discovery in the tongue, the taste receptors, along with several taste signal transduction molecules, have been demonstrated to be expressed in many extraoral locations (e.g., stomach, intestines, liver, pancreas, respiratory system, heart, brain, kidney, urinary bladder, adipose tissue, testis, spermatozoa, lymphocytes, and endocrine glands) [14–21] (see Table 1). Taking into account the wide tissue distribution of taste receptors, a surprisingly strange conclusion arises: the whole body is endowed with taste receptors. At the origin taste receptors were chemoreceptors. Wherever in a biological organism the perception of certain chemicals is necessary, the existence of such receptors is mandatory. What at first glance seemed astonishing (taste receptors everywhere), at further scrutiny appeared as self-evident and necessary (chemoreceptors anywhere they are needed).

This review is intended to summarize and discuss the significance of extraoral taste receptors and other chemosensory processors for ayurvedic pharmacology.

## 2. Taste Reception and Moreover

Sweet taste receptors are heterodimers of the G protein-coupled receptors (GPCR), T1R2, and T1R3 [149]. A wide range of natural or artificial sweet tastants, from simple six-carbon saccharides to guanidinoacetic acids, large peptides, and polypeptides, bind to this single T1R2/T1R3 dimeric receptor [150]. T1R3 subunit has been also shown to form homodimers (T1R3/T1R3) that bind monosaccharides and disaccharides [151], as well as heterodimers (T1R1/T1R3) that bind L-amino acids, mediating the so called “umami” taste. Bitter tasting compounds are detected by receptors belonging to the T2R family of receptor proteins. There are only approximately 25 different T2Rs [152], which detect more than 800 bitter tasting compounds [153, 154]. This is possible because certain T2Rs have a low selectivity (they are more promiscuous, having a broad receptor repertoire or breadth of tuning) [155–157]. Sweet, umami and bitter taste receptors share a common transduction mechanism based on activation of the heterotrimeric G protein, whose *β*/*γ* subunit further conveys the signal for membrane depolarization and generation of an action potential [3, 158].

Ayurveda classifies meat taste as sweet, although modern science classifies it as umami (the Japanese word “umai” means “meaty”); therefore within ayurvedic framework umami should be considered as a peculiar sweet submodality [159]. Interestingly, several scientific findings support the ayurvedic perspective: (1) there are important structural similarities between sweet (T1R2/T1R3) and umami (T1R1/T1R3) taste receptors, both heterodimers, having one subunit in common [160]; (2) mice perceive synergistic umami mixtures (glutamate and ribonucleotide) as tasting sweet [161]; (3) taste cells coexpress the sweet taste and umami taste receptor subunits (all three T1R subunits) [162].

Ayurveda classifies also fats (e.g., clarified butter or ghee, marrow fat, and the majority of oils) as having sweet taste [163]; therefore within ayurvedic framework the newly proposed “fatty taste” should be considered as another peculiar sweet submodality. Several studies showed that tastants eliciting fat taste, like free fatty acids (FFA), may be detected by specific GPCR (e.g., GPCR120) [164–166] and a rather unusual gustatory detector, CD36 (i.e., cluster of differentiation 36), a multifunctional versatile ancestral protein, widely distributed in the body (microvascular endothelium, macrophages, dendritic cells, microglia, retina, erythroid precursors, platelets, liver, adipose tissue, heart, skeletal muscles, breast, kidney, and gut) [167, 168]. These two lipid sensors are coexpressed, probably in type II taste bud cells, and cooperate in fat detection [168, 169]. CD36 displays a greater binding affinity for long chain fatty acids (LCFA) than GPCR120, having the primary role in fat detection, and its expression is downregulated during a meal, in contrast with GPCR120 expression, which is not changed during the meal [170]. The signaling cascade induced by LCFA in taste bud cells showed several similarities with the signal transduction cascade specific for sweet, bitter, and umami taste: GPCR involvement, activation of phospholipase C, calcium signaling, and transient cell depolarization are caused by the opening of the Na^+^-permeable channel called transient receptor potential melastatin-5 (TRPM5) [168, 171, 172].

“Fatty taste” perception via CD36-GCCRs pathway is not the single perception modality. It seems that dual, complementary mechanisms are involved in the detection of dietary fats: (1) a high-sensitivity specifically tuned mechanism (CD36-GCCRs pathway), located in the gustatory epithelium, is involved in the detection of low concentrations of LCFA present in food items or released from triglycerides by a lingual lipase, (2) a low sensitivity, broadly tuned mechanism, represented by the trigeminal pathway, is located in the nongustatory epithelium, involved in the detection of high concentrations of various types of FFA [168].

Astringency is not recognized as a distinct taste, its perception being possible with nontaste oral tissues, and increased with repetitive sampling (a characteristic typical for trigeminal sensation, not for taste sensation) [12, 173, 174]. The most widely accepted definition is that astringency is a long lasting sensorial experience of drying, puckering, or roughness on the tongue and oral cavity, produced by certain food and beverages, most of them rich in tannins, like unripe fruits, nut skin, cocoa, green tea, grape seeds, and red wine [175]. Other compounds able to produce astringent sensation are metal salts (e.g., aluminum ammonium sulfate, aluminum potassium sulfate), acids (e.g., tartaric acid), and dehydrating agents [176]. Scientists explained most often astringency as a trigeminal orosensation: astringent compounds are detected by trigeminal sensors and activate a G protein-coupled signaling pathway that involves recruitment of adenylate cyclase, followed by the activation of cyclic nucleotide-gated channels, and does not involve transient receptor potential (TRP) channels [12]. The astringent signal amplification takes place by Cl^−^ efflux through Ca^2+^-activated Cl^−^ channels in the trigeminal neurons [177]. A possible synergism between a chemosensory and mechanosensory activation of trigeminal sensors was suggested, but this is still under debate and requires validation [177]. The precipitation of salivary proteins by food tannins, especially proline-rich proteins, followed by stimulation of oral mechanosensors [178], as contributing mechanism to the astringency perception, is more or less accepted by the scientists today [12].

Salty and sour are recognized as “mineral taste,” both being evoked by elemental ions (salty taste by Na^+^ concentrations from 10 mM to 500 mM, while sour taste by acidic pH and also weak organic acids, able to permeate the membrane) [3].

Regarding salty taste, the precise transduction mechanisms responsible for this taste and their location remain still unclear [179]. It is not clarified yet whether type I or type III cells are the principal actors in salty taste detection [6–8, 180].

Appetitive responses to NaCl (<100 mM NaCl, called “low salt”) have been linked most likely to amiloride-sensitive epithelial sodium channels (ENaC), while aversive responses to high-salt (>300 mM, referred as “high-salt”) have been correlated with the recruitment of the two primary aversive taste pathways by activating the sour- and bitter-taste-sensing cells and are considered to be amiloride-insensitive [6, 181].

ENaC are expressed on type I taste cells of taste buds [7, 8]. The classical taste “receptor,” in case of appetitive salty taste, is actually a specific transport pathway that allows selectively the Na^+^ and Li^+^ cations (and not other monovalent cations) to enter the taste bud cell and afterwards to spread depolarizing current [182]. ENaC was first proposed to play a role in salty taste over 30 years ago [182]. Scientists know today that ENaCs located in the apical membrane are essential for salty taste perception, but also basolateral channels may contribute [2]. Four homologous epithelial Na channel (ENaC) subunits (*α*, *β*, *γ*, and *δ*) have been identified in mammals. All four ENaC subunits were identified in human taste bud cells as well as in nonchemosensory lingual tissue [183]. The main ENaC is a heterotrimeric assembly of *α*, *β*, and *γ* subunits, characterized primarily by its high affinity for amiloride, a potassium-sparing diuretic which acts precisely by blocking ENaC. The tissue distribution pattern of ENaC isoforms is different, *δ* subunit being expressed mainly in nonepithelial cells, in the brain (cerebellum, cerebral cortex, hippocampus, caudate nucleus, and putamen), pancreas, liver, testis, and ovary, whereas *β* and *γ* subunits mainly in the epithelial cells, in the kidney, lung, and colon [27, 38]. It is not yet clear whether ENaC*δ* is functional in vivo in association with other subunits or active as monomer [27].

High-salt response is nonselective (the detector recognizes a wide range of salts, e.g., Na^+^, K^+^, NH_4_^+^, Ca^2+^, etc.) and it was proposed to be TRPM5/PLC*β*2 dependent in bitter-, as well as in sour-sensing cells [181, 184].

Regarding the sour taste, the molecular identity of sour receptor is still unknown [185]. Although several candidates for sour receptors or transducers have been proposed, including acid-sensing ion channels (ASICs), hyperpolarization-activated cyclic nucleotide-gated (HCN) channels, and transient receptor potential (TRP) channels (polycystic kidney disease protein-like, PKD2L1, PKD1L3); there is no strong evidence of a direct link between these various channels and sour taste transduction [186–188].

Only recently, scientists have discovered that the proximate stimulus is intracellular acidification in type III cells, not extracellular protons per se [4]. Two potential mechanisms mediate this acidification of cytoplasm: (1) a proton influx through a Zn^2+^-sensitive proton conductance (in case of extracellular partly dissociated organic acids, strong inorganic acids) [185]; (2) permeation of protonated organic acids (e.g., acetic acid) into the type III cell cytosol, followed by their dissociation [4]. The consequent drop in the intracellular pH blocks the resting K^+^ current in sour taste cells by triggering the 2-pore-domain potassium channel (K_2_P) [189, 190], and this event ultimately leads to an action potential [185]. This signaling pathway explained why weak acids (which can diffuse across the membrane) taste sourer than strong acids (which cannot diffuse across the lipid bilayer) [191].

Unexpectedly, this sensitivity to intracellular acidification is attributed to relatively ubiquitous ion channels, K_2_P, whose distribution is not restricted to sour taste cells, being expressed in a wide variety of tissues and organs: brain, sperm, heart, kidney, liver, vascular smooth muscle cells, skeletal muscle, and so on (see Table 1) [25, 54, 185]. Considering that such a diversity of cell types might detect acid stimuli, scientists have already raised the question of potential physiological roles of acid-sensitive receptor cells outside of the taste system [191].

K_2_P family contains several members, which play essential background roles in cells, such as control of cellular excitability, volume, and growth [192, 193]. The expression of various K_2_P members in taste bud cells surrounding nontaste epithelium and extraoral locations varies among species [189, 192]. Some members are highly sensitive to intracellular acidification, while others more to extracellular acidification [189, 194].

Pungency or spiciness is a trigeminal sensation, like astringency, not being recognized as a distinct taste in modern medicine, rather belonging to chemesthesis. Chemesthesis is a chemical sense, as well as taste, but refers to the more general sensitivity of the mucous or cutaneous surfaces, perceived as pungency, irritation, but also thermosensations (cooling or heat) [1]. Ayurveda includes under the pungency* (katu rasa)* many sensations that belong to the chemesthesis, such as irritation induced by hot chili peppers (capsaicin), aromatic sensations induced by spices or plants rich in volatile oils (e.g., oregano, mint) [1, 109]. Certain members of the transient receptor potential (TRP) channels family are key players in the perception of pungency: TRP vanilloid types 1, 3, and 4 (TRPV1, TRPV3, and TRPV4) and TRP ankyrin type 1 (TRPA1) for “hot” pungency, while TRPM8 for “cold” pungency [1, 123, 134, 141].

TRP channels are nonselective cation channels permeable to Ca^2+^, Na^+^, Mg^2+^ ions, and so on. The complexity of TRP functionality is far from clear; many studies consider the involvement of TRP also in other taste perception, such as salty, sweet, bitter, detection of temperature, or mechanoreception [1, 195].

It is interesting to notice that the so called “cold” ayurvedic* rasa* (sweet, bitter, and astringent) are detected by receptors coupled with G proteins, while the “hot”* rasa* (salty, sour, pungent), which have a sensorial characteristic of sharpness in Ayurveda, are directly mediated through various types channels, which are penetrating through membrane.

## 3. Extraoral Distribution of Taste Receptors

More and more evidence shows that the localization of taste receptors is not restricted to oral cavity, not even to certain tissues/organs, but rather evenly distributed over the entire body. Even if not all extraoral sites express TRs at levels comparable to taste tissue, this wide distribution suggests that TRs may have functional roles far beyond the original concept of taste perception. It is highly suggestive that a very important study published by Yamamura et al. showed that ENaC*δ* isoform is expressed in all the human tissues tested (more than 50), although in very different proportions [27]. Also TRPs, which are responsible for oral nongustative chemosensitivity, have a wide tissue distribution, like taste receptors. For instance, TRPV1 mRNA was detected in all the tested human tissues [41, 42].

**Figure 1:**
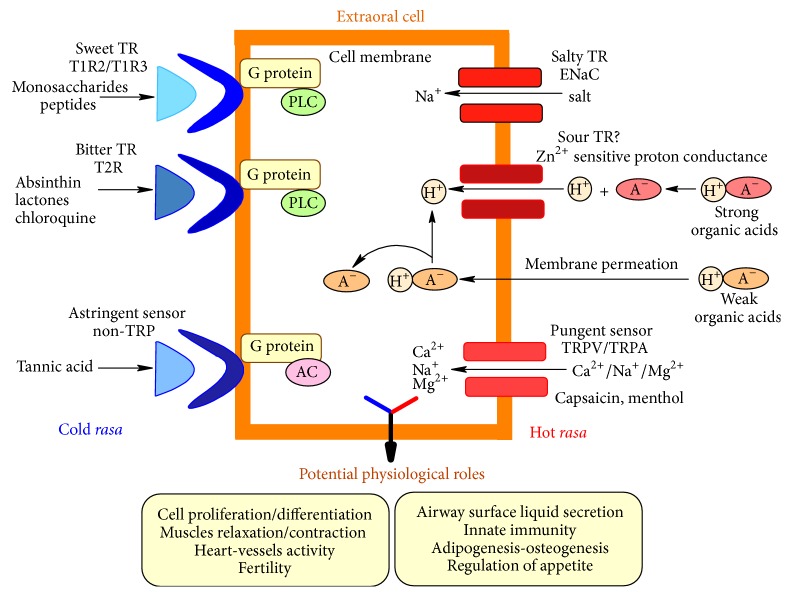
Potential physiological roles of extraoral taste receptors or other chemosensory processors (A^−^: anion, AC: adenylate cyclase, ENaC: epithelial Na^+^ channel, PLC: phospholipase C, TRP: transient receptor potential channel, TRPV: TRP vanilloid type, TRPA: TRP ankyrin type, TR: taste receptor).

Scientists suggested that these extraoral taste cell-related elements that are mainly solitary or clustered cells, not grouped in buds, may be part of a large* diffuse chemosensory system* (DCS), compared with an iceberg, the taste buds representing only the most visible portion, while the extraoral taste cells are the larger “submerged” part [196]. Scientific studies showed that DCS may have crucial physiological roles. DCS seem to be involved in detection of irritants, control of airway surface liquid secretion, innate immunity, microbial population, regulation of appetite, cell proliferation, relaxation/contraction of muscles, bronchia, urinary bladder, and vessels, and regulation of heart activity (Figure 1) [150, 197–201].

The functional implications of these taste receptors and TRPs widely dispersed in various extragustatory tissues also shed a new light on several ayurvedic concepts used in ayurvedic pharmacology* (dravyaguna vijnana)*, such as taste* (rasa)*, postdigestive effect* (vipaka)*, qualities* (guna)*, and energetic nature* (virya)* of medicinal plants and food.

## 4. Ethnopharmacological Descriptors in Ayurveda

Ayurvedic pharmacological description of a medicinal plant uses a set of* ethnopharmacological descriptors* that are groups of herbal attributes:* rasa*: taste,* guna: *qualities,* vipaka*: postdigestive effect,* virya*: energetic nature/potency,* prabhava*: special/extraordinary potency.

All herbal descriptors are traditionally used to select the medicinal plants for the treatment of various diseases.

In Ayurveda tastes* (rasa)*, two by two, are complementary in terms of qualities* (guna)*; for instance salty taste (which is hot, heavy and wet) antagonizes bitter taste (which is cold, light and dry) (see Table 2) [109].

Further experimental studies are required in order to verify whether these* ethnopharmacological descriptors* may become predictor tools for specific pharmacological activities.

An interesting finding is that the gustatory neurons in the rostral nucleus of solitary tract respond not only to tastant compounds, but also to somatosensory inputs, such as tactile and temperature stimulation, as well [202], explaining the integrative perception of a food item quality, which is also described in Ayurveda by several correspondent ethnopharmacological descriptors (*guna*, e.g., smoothness tactile sensation*, virya, *hot or cold sensation).

## 5. Taste (*rasa*) Concept in Ayurveda

**Figure 2:**
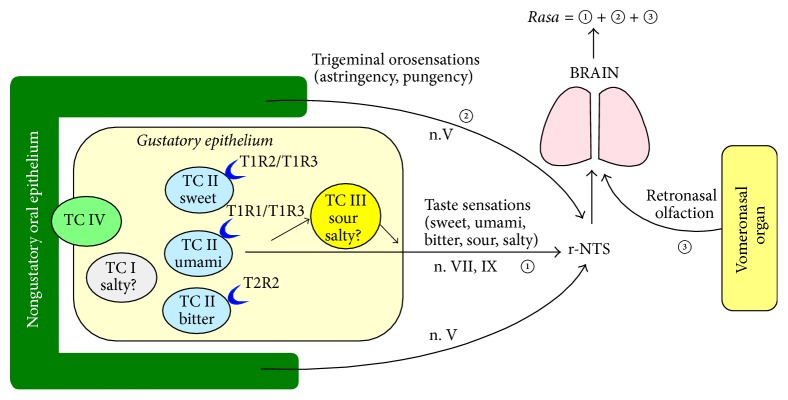
Complex meaning of* rasa* (TC: taste receptor cell, TR: taste receptor, n.V: trigeminal nerve, n. VII: facial nerve/chorda tympani branch, n IX: glossopharyngeal nerve/lingual branch, r-NTS: rostral division of the nucleus tractus solitarius).

The ayurvedic physicians agree that the Sanskrit term* rasa* designates not only the taste or gustatory sensation perceived through the oral taste buds, but also the flavor experienced by retronasal olfaction [203]. Moreover, taking into account the modern definitions of astringency and pungency as trigeminal orosensations, we hypothesize that the ayurvedic term* rasa* has a much more complex meaning, designating, beyond taste and retronasal olfaction, also the trigeminal orosensations (Figure 2).

Regarding the term* rasa, *in the present paper we shall use, as equivalent English term, the word “taste,” for the purpose of simplicity, although this only approximates the versatility of* rasa.*

According to Ayurveda,* rasa* represents an attribute of the substance* (dravya)*, being experienced the moment a substance comes into contact with the tongue [109].

Taste of medicinal plants has been considered for millennia in Ayurveda, as the most important ethnopharmacological descriptor used for identification of drug properties. At the beginning of the chapter on the properties of the drugs, in Cakrapanidatta's commentary to Caraka Samhita (one of the three major ancient Ayurveda texts), it is underlined: “Among* rasa, virya* and* vipaka*,* rasa* is the most important one; hence the discussion in this chapter is initiated with the description of* rasa* (taste).” (Caraka samhita, Sutrasthana XXVI.1-2/Cakrapanidatta's Agnivesa Dipika) [109]. Why is this? One of the reasons would be the fact that* rasa* can always be ascertained by direct perception (Sansk.* pratyaksha*), while* virya *and* vipaka *not (observing the drug action on the body, after ingestion, while the drug is processed through digestion and metabolism, should sometimes be the basis for inferring hot/cold* virya* and should always be the ground for deducing* vipaka*) (Caraka Samhita, Sutrasthana XXVI.66/ Cakrapanidatta's Agnivesa Dipika) [109, 204]. Since direct perception of* rasa*, without any subsequent analysis, represents an unbiased observation (the main requirement of any scientific study),* rasa *is considered the most important tool of drug discovery in Ayurveda [203].

Another potential explanation lies in the relative causality link between different ethnopharmacological categories:

“The substance* (dravya)* is the origin/cause* (ashraya)* and taste* (rasa)* is the effect* (karya)*. (…) Similarly, the attributes* (guna)* and pharmacological actions* (karman)* are dependent upon the taste* (rasa)*, which is the origin/cause* (ashraya)*.” (Caraka Samhita, Sutrasthana XXVI.66/Cakrapanidatta's Agnivesa Dipika) [109].

The importance of ancient science of* rasa* is suggested also by the following fragment: “Even the knowledge of the classification of* rasa alone* may help in the identification of etiology, symptomatology and treatment of diseases.” (Caraka Samhita, Sutrasthana XXVI.27/Cakrapanidatta's Agnivesa Dipika) [109]. In Ayurveda, apart from the humoral classifications of diseases based on three* dosas *(*Vata, Pitta, and Kapha* humors) or hot/cold qualities, there is an alternative classification into six main categories characterized by the excess of a certain* rasa*: excessive sweetness, saltiness, sourness, bitterness, pungency, and astringency [109, 110, 163]. Within this framework of* rasa*, not only the etiology, but also the treatment is* rasa*-oriented.

**Figure 3:**
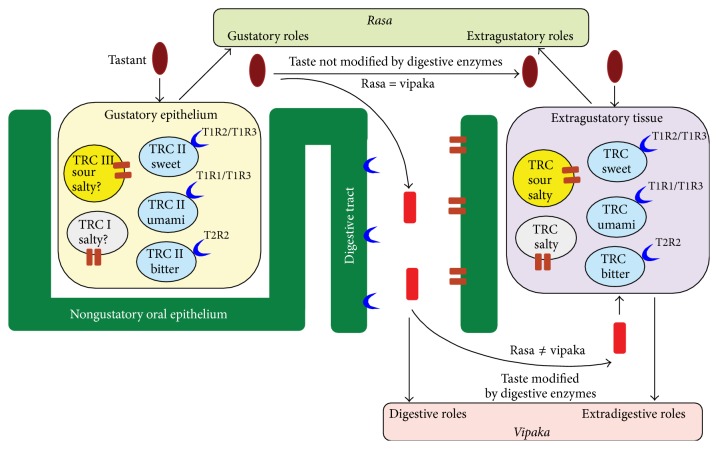
*Rasa *(taste) versus* vipaka* (taste after digestion) (TRC: taste receptor cells, TR: taste receptor).

We estimate that new dimensions of this ancient Ayurveda theory will be revealed in the light of extraoral taste receptor discovery, as their functions will be understood. Even if the extraoral receptors do not play a role in the taste (as this is understood and defined by the modern science), they may play functional roles in connection with* rasa*, which is a broader concept than the modern concept of taste, and which includes both gustative and extragustative roles (Figure 3).

It is worth mentioning that in contrast with Ayurveda, in other traditional medical systems, such as Galen's humoral theory, Greek-Arabic, Aztec, Mayan, and Zapotec medical traditions, “hot,” “cold,” “dry,” and “wet” qualities are more important as descriptors than the organoleptic properties, these four qualities being used to systematically categorize remedies, foods, and diseases [205–207].

Furthermore, in Ayurveda, the* rasa* (taste) of the herbal drugs is not the only descriptor potentially related to the ethnopharmacological activities. This assumption is reasonable, taking into account the fact that some of the pharmacologically active compounds present in a plant, are not necessarily contributing to the specific perceptible herbal taste (although some of them may still contribute to the imperceptible taste, when they are present in minute amounts, see Caraka Samhita, Sutrasthana XXVI.8/Ayurveda Dipika:* “Imperceptibibility [sic] of the taste may arise because of high dilution”* [109]).

Cold/hot properties* (virya)* and postdigestive effect* (vipaka)* are also recognized as important ayurvedic descriptors, as well as causes (Sanskr.* ashraya*) for the ethnopharmacological activities. Moreover, in terms of therapeutic efficacy, in Ayurveda as well, another hierarchical order of descriptors is mentioned. As a rule, medicinal plants display their essential therapeutic activities by virtue of their attribute which possesses the biggest strength (Sanskrit* bala, *strength): certain plants/activities by virtue of their* rasa* (if* rasa* is the most intense herbal property), some by virtue of their* virya* or other qualities (if* virya* or other qualities have the biggest* bala* or strength), some by virtue of their* vipaka* (when* vipaka* is the strongest), and some by virtue of their* prabhava*. Nevertheless, “when* rasa, vipaka, virya*, and* prabhava* are all of equal strength,* rasa* is exceeded by* vipaka*, both of them in turn are exceeded by* virya*, and* prabhava* overcomes all of them.” (Caraka Samhita, Sutrasthana XXVI.71-72) [109]. Remarkably, one recent experimental study on 71 herbal drugs used in Zoque community of healers, Mexico, found that there is a dominance of humoral qualities over chemosensory properties (taste, smell) as predictors of medicinal indications [208], similarly with the previously mentioned therapeutic supremacy of* virya* over* rasa.*

As a rule, properties such as* virya* (hot/cold potency) and* vipaka* (postdigestive effect) are inferred based on the perceived taste (*rasa*) (Table 1) [109, 204]. In case of medicinal plants where “*virya* and* vipaka* are in conformity with* rasa*, the pharmacological activities are explained in terms of* rasa* only” (Caraka Samhita, Sutrasthana XXVI.46-47/Ayurveda Dipika) [109].

Ayurveda texts mention also that there are remedies which do not follow the inference rule, and whose* virya* is contradictory to* rasa* (Caraka Samhita, Sutrasthana XXVI.48-49) [109]. Example of such exceptions from the inference rule are represented by amalaki* (Emblica officinalis)*, sour and cold, and daruharidra or Indian barberry* (Berberis aristata)*, bitter and hot [119]. Such cases are explained on the basis of* prabhava*, implying the specific unique chemical composition of the plant.* “Prabhava (specific action) is nothing but the inherent active principle of the remedy*.*”* (Caraka Samhita, Sutrasthana XXVI.68-72/Ayurveda Dipika) [109].

Several scientific studies showed that there are statistically significant associations between the organoleptic properties of medicinal plants (taste, smell) and their ethnomedical indications or activities [208–210].

We have found in a recent Ayurveda literature study, by mapping the ayurvedic ethnopharmacological space, that there are statistical associations between various tastes and certain therapeutic activities in Ayurveda system [159]. The ethnopharmacological activities, which are traditionally considered to be more representative for a specific taste, appeared in our study to have higher statistical power: bitter–digestive, detoxifying, and antipoisoning, sweet–nutritive tonic/weight promoter, body strengthening/invigorating, and spermatopoetic, pungent-aperitive, beneficial for throat, astringent–antidiarrheal, and so on. [159]. Nevertheless, these results did not allow us to conclude whether* rasa* is a true predictor of herbal therapeutic activities in Ayurveda, or the therapeutic activities were assigned a posteriori, according to the herbal perceived effectiveness in the treatment of certain diseases. Therefore, this kind of results should be carefully interpreted and validated by further experimental studies.

The discovery of the extraoral location of taste receptors marked the beginning of a new era not only for sensory research and nutritional science [14], but also for ethnomedicine, providing now a new perspective and framework for understanding specific ethnomedical epistemology, including Ayurveda concepts and methods.

The extragustatory taste receptors are already recognized nowadays as potential drug – targets, as well as phytochemical targets, useful for the treatment of several diseases, such as obesity, type 2 diabetes, hyperlipidemia, asthma, infertility, immunity disorders, anxiety, pain, cancer, and autoimmune disorders [150, 160, 211]. Thus, the ancient claim of Ayurveda concerning the systemic action of* rasa* (taste) throughout the body might be reinterpreted in the light of this new discovery.

We shall discuss in the following part of the paper certain similarities between Ayurveda knowledge on* rasa* and modern scientific information on taste, arising from the recent scientific studies.

## 6. Traditional Taste (*rasa*) in the Light of Modern Science

### 6.1. Sweet Taste (Sanskr.* madhura rasa*)

*Nourishing and promoter of growth *(Sanskr.* Brimhana*). Ayurveda declares that sweet taste nourishes all the seven bodily tissues (*rasa*: plasma, lymph, and leukocytes,* rakta*: erythrocytes,* mamsa*: muscles,* meda*: adipose tissue,* asthi*: bone,* majja*: marrow and nervous tissue,* shukra*: semen) [109].

Scientists have already discovered that sweet receptor is involved in insulin secretion: (a) indirect mechanism–activation of sweet receptor transduction pathway in enteroendocrine L cells led to the release of glucagon-like peptide-1 (GLP-1), which in turn enhanced insulin release from the pancreas [212]; (b) direct mechanism–sweet taste receptor expressed in pancreatic *β*-cells stimulates insulin secretion [36]. Insulin is recognized as the principal anabolic hormone in the body and regulator of mammalian target of rapamycin complex (mTORC), which activates cell growth and protein translation and suppresses autophagy [213]. Insulin-like growth factors (IGF) has a central role in mediating trophic hormone action in many tissues (bone, cartilage, muscles, intestine, and neurons) [214, 215].

Concerning erythrocytes* (rakta)* and neurons* (majja)*, it is also well known that they are glucose dependent (“sweet taste dependent”) cells, requiring glucose for their energetic needs and survival. Moreover, mice lacking the T1R3 taste receptor gene developed impaired glucose metabolism, indicating possible involvement of T1R3-mediated glucose sensing mechanisms in the brain [216]. Ischemia caused by blood restriction in the brain enhanced sweet taste receptor expression in reactive astrocytes, a potential protective mechanism against neuronal “sweet” deprivation [217].

**Figure 4:**
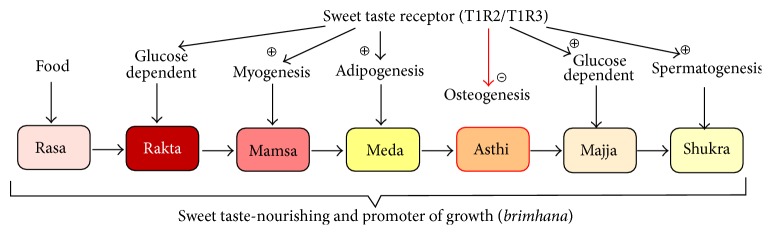
Interferences of sweet taste and sweet taste receptor with tissue* (dhatu)* generation cycle (in Ayurveda the tissues are generated in a successive order:* rasa, rakta, mamsa, meda, asthi, majja, and shukra*.).

Modern medicine showed that sweet receptor (T1R2/T1R3) is also involved in the tissue renewal from stem cells or precursors. For instance, sweet receptor activation stimulated adipogenesis, mediating differentiation of preadipocytes into adipocytes [21, 150]. T1R2−/− knockout mice had decreased numbers of adipocytes in the bone marrow microenvironment [218]. Muscle regulatory factors (e.g., myogenin) might control myogenesis and skeletal muscle metabolism through the regulation of T1R3 expression [219]. T1R3−/− knockout mice showed reduced degree of mTORC1 activation and increased rate of autophagy in the skeletal muscle, suggesting the role of T1R3 in tissue nutrition and normal development [220]. Similarly, in Ayurveda, sweet taste* (madhura rasa)* is considered to be useful for the physiological tissue growth and regeneration of skeletal muscles* (mamsa)*, adipose tissue* (meda)*, and marrow and nervous tissue* (majja)*. Although, we have to mention that there is contradictory information about Ayurveda versus modern science, in terms of bone growth. Sweet taste promotes bone development according to Ayurveda, while it inhibits osteogenesis, according to research studies (Figure 4) [150].

*Spermatopoetic (Sanskr. Shukrajanana)*. Sweet taste transduction is required for sperm development and maturation, while its blockade causes male infertility in animals [85]. Expression patterns of T1R3 and G*α* in the mice testis during various stages of life and throughout the spermatogenic cycle showed that T1R3 and G*α* may play important roles in the onset of spermatogenesis, rhythm of spermatogenic cycle, and ageing of the testis [221]. T1R signaling cascade in mammalian spermatozoa also controls the sequential process of fertilization via regulation of basal Ca^2+^ and cAMP intracellular concentrations, thus maintaining spermatozoa in a quiescent state in the female reproductive tract until they reach the oocytes [222].

Scientists estimated that even low levels of environmental chemicals (e.g., phenoxyauxin herbicides) or drugs (e.g., lipid-lowering fibrates) that are T1R3 (sweet taste receptor) inhibitors could lower sperm count and negatively influence human male fertility, while activators of sweet taste receptors may help male fertility [85, 223].

Similarly, in Ayurveda, sweet taste is useful for sperm* (shukra)* growth [109]. 

*Antiageing (Sanskr. Vayasthapana)*. There are contradictory statements, in terms of sweet taste effects on ageing. Sweet taste promotes longevity, according to Ayurveda [109], but it seems to decrease lifespan according to modern science [224]. Nevertheless there is no real contradiction if the facts are put in the appropriate context. Sweet taste in its broadest ayurvedic meaning encompasses practically all nourishing substances, not only simple and complex carbohydrates, but also proteins and fats. In rich countries excessive alimentation (i.e., an excess of ayur-sweet taste) indeed endangers health, promotes disease (diabetes, cancer, and cardiovascular disorders, to mention only a few), and therefore curtails life. However in underprivileged regions of the world proper alimentation (i.e., adequate quantities of ayur-sweet taste) is an essential health-maintaining and consequently life-prolonging agent.

Two of the most important discoveries in the field of ageing research seem to contradict Ayurveda theory on sweet antiageing effects: inhibition of gerontogenes* daf-2* and* age-1* that encode two elements of the insulin/IGF-1 signaling pathway, dramatically extended lifespan in* C. elegans *[225, 226]. Also deletion of calcium homeostasis modulator 1 (CALHM1), a voltage-gated ion channel involved in sweet taste reception, prolonged the lifespan in animal model [224]. In other words, a reduced reception of sweetness, as well as reduced activation of insulin pathway (which normally is activated by sugars), would be beneficial for longevity.

Nevertheless, other studies reveled opposite effects of sweet taste: in fruit flies* (Drosophila melanogaster)*, sweet taste receptor (Gr5a) mutants had a shorter lifespan, whereas bitter taste receptor mutants (GR66a) have a longer lifespan [227]. Therefore, the ability to taste sweet has positive effects on longevity, similarly with Ayurveda claim, while the ability to taste bitter taste could have negative effects on lifespan in fruit flies.

Also, T1R3 expression varies along the life. Sweet taste receptor (T1R3) expressions in mouse testis increased significantly from prepubertal to pubertal periods, and decreased significantly in aged animals [221]. Older animals showed reduced sweet taste responsivity compared with the younger animals due to a significant decrease in the number of taste cells expressing T1R3, while the other taste modalities (salty, sour, and bitter) were not affected by ageing [228]. Further studies should clarify whether these changes in sweetness reception represent only an epiphenomenon of the ageing process, or may be a contributing factor to ageing.

Concerning the sweet plants or phytochemicals, there are isolated reports on their antiaging potential. Licorice* (Glycyrrhiza glabra)*, one of the most intensely sweet medicinal plants, is found in the top 10 botanical ingredients of antiaging creams [229].

*Diabetogenic Activity*. Sweet taste receptors expressed in gut and other endocrine organs may have an important role in glucose metabolism and their altered activity may contribute to type II diabetes and obesity pathogenesis [223]. When excessively consumed, sweet taste induces obesity (sanskr.* meda roga*) and diabetes (sanskr.* madhumeda*), according to Ayurveda also [109]. 

*Limitations of the Present Hypothesis on Extraoral Taste Receptors as Molecular Basis for Sweet Taste (Madura Rasa) Ethnopharmacological Activities*. Sweet compounds may have pharmacological activities that are not mediated by their taste* (rasa)* attribute. For instance, artificial sweeteners stimulated adipogenesis and suppressed lipolysis independently of sweet taste receptors T1R2/T1R3 [230]. Another limitation is represented by the fact that certain sweet tastants may have pharmacological activities which are not traditionally assigned to sweet* rasa*, or they are assigned to other* rasa*, for example increased contractility of the bladder induced by artificial sweeteners [49], antiviral activity of glycyrrhizic acid (the phytochemical responsible for the sweet taste of licorice) [231].

### 6.2. Bitter Taste (Sanskr.* tikta rasa*)

*Antiadiposity (Sanskr. Medoshoshana). *Bitter tasting medicinal plants and food items “dry up” the adipose tissue (Sanskr.* meda*) and muscle fat (Sanskr.* vasa*), according to Ayurveda [110]. Regarding this bitter taste ethnopharmacological activity, modern science has a similar perspective. Avau et al. showed that the treatment of high fat fed obese mice with bitter agonist quinine resulted in an *α*-gustducin-dependent decrease in body weight associated with a decrease in food intake. This effect is probably mediated by bitter taste receptor (T2R), via decreased differentiation of preadipocytes into mature adipocytes [232]. 

*Antitoxic (Sanskr. Vishaghna).* In Ayurveda, bitter taste is considered antitoxic* (vishaghna)* [109]. The traditional concept of “toxin” is quite complex, versatile, and difficult to define in modern medical terms. Ayurveda states that there are two main types of toxins* (visha)*:* amavisha* (endogenous toxins or metabolic toxins resulted from a defective tissue metabolism,* ama *means “unripe”) and* garvisha* (exogenous toxins or environmental toxins that include spoiled food, chemicals, pollutants, heavy metals, etc.) [110, 233].

Bitter taste may sometime represent a signal of toxic substances that may be harmful (e.g., certain alkaloids, rancid fats, microbial fermentation derived compounds, and certain environmental chemicals), while bitter sensing may act in these circumstances as a repellent mechanism for noxious substances [234, 235]. The presence of a large group of T2R bitter sensors with a broad receptor repertoire or breadth of tuning was proposed as a necessary evolutionary feature in order to minimize failure in detecting bitter toxins. Scientists have also proposed that, for the same reason, one single bitter substance can stimulate up to 15 different human T2Rs [155].

Interestingly, bitter tasting chemicals (e.g., 6-n-propyl-2-thiouracil, denatonium benzoate, and phenylthiocarbamide) delayed gastric emptying via ghrelin, decreased long term food intake, or induced fluid secretion in colon [32, 236, 237]. These mechanisms would potentially either prevent the ingestion and absorption of poison from the small intestine or contribute to the flushing out of poisons from the gut [32]. From an ayurvedic perspective, 6-n-propyl-2-thiouracil, denatonium benzoate, and phenylthiocarbamide, apart from being bitter, belong to the* garvisha *type of toxin, and their effect on the gastrointestinal motility may be interpreted as a* vishagna* protective mechanism.

On the contrary, salicin, also a bitter taste receptor agonist, but a natural one (not identified as* garvisha* by the body), had opposite effects to those elicited by the previously mentioned synthetic agonists: salicin increased food ingestion (ayur-bitter is* rucikara *and increases appetite [110]) and accelerated gastric emptying [236]. Unfortunately, these studies do not prove the roles of extraoral bitter taste receptors as mediators of these activities, being only parallel studies.

Also, bitter rejection response is not always adaptive, since there is no direct relationship between toxicity and bitter taste thresholds [238]. Scientists eventually concluded that heightened perception of bitterness, which has a genetic basis, is actually one common reason for bitter rejection [239–241].

On the other side, bitter avoidance on the long term may be detrimental to health, since many bitter phytonutrients (e.g., polyphenols, flavonoids, isoflavones, terpenes, and glucosinolates) appear to lower the risk of cancer and cardiovascular disease [234, 242].

Cancer and cardiovascular disease are considered to be pathological states associated with accumulation of* amavisha *(metabolic toxins).

Cancer cells are glucose addicted and set on anaerobic glycolysis (Warburg effect) [243], which causes accumulation of lactate. Since glucose is not completely oxidized in anaerobiosis, lactate might be considered an “unripe” product of metabolism, which is, according to Ayurveda, an endogenous toxin* (amavisa)*. Lactate is considered nowadays an insidious metabolite, which alters several cellular pathways, and ultimately contributes to immunosuppression, tumour metastasis, and tumour growth [244]. Scientists have proposed that targeting the Warburg effect may be a new therapeutic anticancer strategy [245]. Many bitter phytochemicals (e.g., morin, scutellarein, naringenin, quercetin, and hesperetin) showed the capacity to counteract Warburg effect, by induction of a metabolic reprogramming of cancer cells, through downregulation of glucose transporter 1 (GLUT1) and inhibition of glycolysis (via hexokinase 2, pyruvate kinase M2, and lactate dehydrogenase A), leading eventually to apoptosis [246–251]. All these effects may be included under* vishagna* activity, which is assigned to bitter tasting medicinal plants or food in Ayurveda.

Bitter compounds (e.g., isothiocyanates and glucosinolates from broccoli and cauliflower, naringin from grapefruit) inhibited the activation of carcinogens (a type of* garvisha*) by phase 1 enzymes (cytochrome P-450) and/or accelerated detoxification of carcinogens by inducing phase 2 enzymes [239, 242], therefore displaying the antitoxic* (vishagna)* property according to Ayurveda.

Another example of* amavisha* accumulation is represented by the cardiovascular diseases, which are associated with a state of oxidative stress and accumulation of lipid peroxides, oxidized proteins, advanced glycation end products [252, 253], and potential forms of* amavisha. *Cardiovascular protection exerted by certain bitter compounds with antioxidant activity may be also interpreted as a type a* vishagna* defensive mechanism.

Whether the extraoral bitter taste receptor directly contributes or not to the anti-Warburg effect, accelerated detoxification and antioxidant protection remain to be elucidated. 

*Anti-Infectious (Sanskr. Krimighna)*. Scientific studies have proven the role of certain secondary metabolites in plant response to pathogenic microorganisms or defense against herbivores. Although these are commonly considered as plant antibiotics, recent studies also suggest their potential involvement in controlling plant immune responses [254]. Many of these compounds are bitter tasting: terpenoid toxins and alkaloids. The antibiotic role of bitter compounds could therefore be interpreted in the context of chemical ecology. Nevertheless, this perspective has certain limitations, since not only bitterness, but also astringency (tannins), sourness (organic acids), and even sweetness (mannose) may be used in plant defense [255].

This association of bitter taste with anti-infectious activity might also have a physiological basis. Several studies showed that bacterial compounds (e.g., acyl-homoserine lactone, N-(3-oxododecanoyl)-l-homoserine lactone) as well as certain bitter phytochemicals (absinthin from* Artemisia annua* or wormwood) activate the bitter taste receptor transduction pathway in respiratory tract and leukocytes, leading to an increased production of antimicrobial peptides [256] and nitric oxide (that also has direct bactericidal activity), increased mucociliary clearance in respiratory tract [257], chemotaxis, up-regulation of CD11b expression, and enhanced phagocytosis [82]. T2R detects bacterial quorum-sensing molecules (e.g., lactones) and may prevent bacterial biofilm formation also [256]. T2R38 supertaster genotype plays a role in sinonasal and gingival innate immunity, by several mechanisms: stimulation of nitric oxide production, increased ciliary beating, direct killing of bacteria, and ability to induce a high level of antimicrobial compounds (e.g., peptide hBD-2). All these aspects suggest that T2R38 may contribute to the protection against upper respiratory infection, chronic rhinosinusitis, and caries in this genotype [258, 259]. Thus, bitter taste receptors are more and more considered as regulators of innate immunity.

Ayurveda, also, states that bitter taste is anti-infectious* (krimighna)*, many of the medicinal plants traditionally used for the treatment of infections having bitter taste (e.g., neem or* Azadirachta indica*, bhumyamalaki or* Phyllanthus niruri*, katuki or* Picrorhiza kurroa*, etc.) [109]. A statistical analysis in a database containing 460 Indian medicinal plants mentioned in Ayurvedic Materia Medica showed us a significant association between bitter taste (tikta rasa) and traditionally assigned anti-infectious activity (*krimighna)* (*p* < 0, 05, OR = 1,13–2,7). Apart from bitter taste, only pungency, among the six* rasa,* was also found to be statistically associated with anti-infectious activity* (krimighna)* (see Pungency).

*Antiasthmatic (Sanskr. Svasahara).* Bitter tastants also caused relaxation of isolated human airway smooth muscle and bronchodilation in a mouse model of asthma, an effect that was greater than that induced by *β*-adrenergic receptor agonists [260]. Interestingly, we have found in a recent study performed on a database of medicinal plants, a positive association (*p* < 0.01, OR 2.966) between bitter ayurvedic herbs and* svasahara* (engl. “which relieves asthma”) activity, although this association is not mentioned as such in the traditional texts [159]. Nevertheless, this finding requires further confirmation in experimental studies. 

*Antifertility (When Used in Excess). *It is well known that animals that can avoid bitter tasting foods are more fertile, generating higher quality spermatozoa and producing more progeny [261].

Since numerous potentially noxious chemicals evoke bitter taste [234, 235, 262, 263], an evolutionary hypothesis on taste and human reproduction claims that the increased sensitivity to bitter stimuli and feelings of nausea in response to bitter foods in pregnant women might represent a protective mechanism during the time of fetal organogenesis, when the major fetal organs are highly sensitive to low levels of toxins [29–31]. Arguments invoked to support this hypothesis are offered by several studies on pregnant women, which showed that nausea during the first trimester is associated with a reduced risk or miscarriages and with a higher chance to have healthier newborns with a bigger birthweight [264–268].

According to the ayurvedic perspective, when used in excess, bitter taste also has harmful effects on fertility, depleting “semen”* (shukra)* and ovum* (arta)*, among other tissues [109]. One recent study on transgenic mice and bitter taste receptor T2R5 seems to contradict Ayurveda theory. It has shown that depletion of T2R5 resulted in smaller testes and led to male infertility [86]. Bitter tasting ability is necessary for fertility, due to several reasons: (1) T2R may be directly involved in spermatogenesis; (2) bitter sensing could be used to detect (both beneficial or potentially noxious) bitter chemical elements in the lumen of the seminiferous tubule. Taking into account the fact that Ayurveda states that* only* excessive bitter taste depletes semen, not the normal amount of bitter, the apparent contradiction disappears (Caraka Samhita. Sutrasthana. XXVI.43-v) [109]. Why? Because the excessive amount of bitter tastants may induce a downregulation of bitter taste receptor expression, which may disturb normal spermatogenesis, similarly with the effect of depletion of bitter taste receptors.

Moreover, T2R5 are expressed in the spermatid phase, but not all spermatids express T2R5 [86]. What was more surprising was the fact that the normal spermatogenesis was maintained after T2R5+ cell ablation; therefore T2R5, although important, is not absolutely essential for spermatogenesis [14, 86]. Nevertheless, bitter taste receptors have versatile modulatory activity in reproduction, and the various aspects of their function remain largely to be determined [269]. 

*Other Potential Activities*. Science does not know the functions of bitter taste receptors which are expressed in the mammary epithelial cells. Although, scientists suggested that these receptors, which are downregulated in breast cancer cells, may be potential therapeutic targets, since several bitter compounds (chloroquine, quinidine, bitter melon extract, and cucurbitacins B and E) were described as inhibitors of tumour growth and proapoptotic agents in cancer cells [94]. According to Ayurveda, bitter taste acts also in the breast, by “purifying” the milk* (stanya)* and the mammary gland channels* (stanyavaha srota)* [109]. 

*Limitations of the Present Hypothesis on Extraoral Taste Receptors as Molecular Basis for Bitter Taste (Tikta Rasa) Ethnopharmacological Activities*. Unfortunately, there is no general agreement on the traditional description of bitter taste in different cultures: bitter taste is “cold” in Ayurveda [109], but it is “hot” in Maya [210], and this may result in differences between indications of bitter plants.

There are several weaknesses of our hypothesis. One would be related to the scarcity of data on the functional roles of bitter taste receptors in various extraoral systems in humans (e.g., gastrointestinal tract), the majority of the studies focusing on their role in the respiratory system [269]. Another one consists in the fact that many published papers nourished the wrong belief that homologous mice and human T2R would have a similar profile of agonists [157].

A delicate topic related to this taste is that many synthetic drugs taste bitter [270]. Nevertheless, they have a large variety of pharmacological targets, besides the bitter taste receptors, as well as effects that cannot be explained on the basis of extraoral bitter taste receptors. In many cases, these extrataste pharmacological targets are more likely to mediate the extrataste effects of bitter drugs. Ayurveda also mentioned such cases, when drugs activities may be explained not by* rasa*, but by other attributes. We estimate that these extrataste mechanisms of action might be categorized in Ayurveda mainly as* prabhava* (special potency), the knowledge thereof being unachievable by inference* (anumana)*.

### 6.3. Salty Taste (Sanskr.* Lavana Rasa*)

*Antiarthritic (Sanskr. Stambha Vidhmapana).* Salty taste eliminates rigidity, being characterized in Ayurveda as* stambha vidhmapana* (Sanskr.* Stambha: *rigidity,* vidhmapana: *dispersing). In Ayurveda rigidity (*stambha*) is a characteristic of arthropathies, for instance cervical spondylosis is called* griva stambha*. Remarkably, the expression of ENaC is decreased (“ayur-deficiency of salty taste”) in osteoarthritis [78].

*Deobstruent (Sanskr. Avakashakara). *Salty taste cures obstruction and accumulation according to the ancient traditional texts [109]. The role of ENaCs as mechanosensors may be correlated with this ethnopharmacological activity of salty taste. Various types of obstructions/lack of flow/fluid accumulation were found to be associated with altered ENaC expression, either underexpression (usually responsible for a deficient Na^+^ secretion, therefore impaired fluid flow in excretory channel) or overexpression (commonly responsible for an excessive Na^+^ reabsorption, therefore also impaired fluid flow in the excretory channel). For instance, in animal models with autosomal recessive polycystic kidney disease, scientists found a decreased ENaC expression in cystic epithelium, which seems to contribute to cystogenesis and development of disease [271]. One of the most probable explanations for the cysts formation is the unchecked proliferation of epithelial cells in the kidney tubules (due to defective primary cilia) [272], resulting in outbulgings of the tubules' walls, which continue to grow until they lose connection to the original tubules, trapping fluid inside; the inability of the fluid to flow down the tubules leads to fluid accumulation. Several groups of scientists have already reported the role of ENaC in epithelial cell proliferation [273, 274].

In patients with human bladder outlet obstruction, the expression levels of each ENaC subunit were significantly greater than those in controls, suggesting the implication of ENaC expressed in the bladder epithelium in the mechanosensory transduction, in the bladder afferent pathways [51]. The ENaC family members were proposed as potential targets for the future management of urinary storage symptoms in spinal cord injury [275]. Impaired airway mucociliary clearance in respiratory diseases, such as cystic fibrosis, chronic obstructive pulmonary disease, is also associated with ENaC overexpression and Na^+^ hyperreabsorption, and ENaC inhibition may be a future therapeutic solution [276].

Variants of ENaC were associated with development of bronchiectasis [277], while a dysfunction of cerebrovascular ENaC, due to a genetic variant of a regulatory kinase was found in patients with ischemic stroke [278].

All these studies suggest that normal functioning of salty taste transduction is required in order to prevent urinary, pulmonary, or blood vessel “obstructions” and fluid accumulation, facts in accordance with Ayurveda knowledge. 

*Sudorific and Lubrifying. *Salty taste has sudorific activity (Sanskr.* svedana*), causes salivation, and lubrifies the tissues* (snehana)* according to ancient Ayurveda texts [109]. It is highly suggestive that ENaCs are expressed in both sweat and salivary glands and have functional roles in sweating and salivary production [279, 280].

*Blood Pressure Control*. Ayurveda states that salty taste clarifies the channels of circulation and that the excessive use of salty taste leads to hypertension (Sanskr.* rakta gata vata*) and bleeding from different parts of the body (Sanskr.* raktapitta*) [109]. It is well known that salt taste sensitivity, as well as ENaC expression, are diminished in hypertensive subjects [281]. Whether the excessive ingestion of salt represents the cause or the effect of this low ENaC expression remains to be clarified. Modern science also found that ENaCs (“salty taste receptor”) in the distal nephron, as well as in the colon, control Na^+^ balance, extracellular fluid volume, and blood pressure, being regulated by aldosterone [282]. Liddle's syndrome (pseudoaldosteronism), a rare genetic disease characterized by extreme hypertension, is caused by mutations in ENaC subunits, leading to urinary salt retention [283].

Excessive dietary saltiness induces negative effects according to modern science, these effects being almost completely predicted by Ayurveda (Table 3).

### 6.4. Sour Taste (Sanskr.* Amla Rasa*)

In Ayurveda fermented foods and beverages like cheese, yogurt, kefir, buttermilk, pickles, fermented sausage, vinegar, alcoholic beverages, fermented soybean, and so on are considered to have a sour taste component. In modern nutrition as well, acids and sour taste are markers of fermentation, being appreciated by humans around the globe [268]. For a list of traditional fermented food items in different culinary cultures, items which are still popular today, see [284]. Carbonated drinks have also a component of sour taste (due to carbonic acid generation during carbonatation process) [285].

Another source of sourness in the modern diet is represented by the food acidulants (e.g., acetic acid, citric acid, fumaric acid, tartaric acid, etc.). Sour taste is the key element in the flavor profile of most acidulants, which are frequently used in food industry for various purposes: to increase flavor, to inhibit microbial growth in food products, to chelate heavy metal ions, to augment the effect of antioxidants, to prevent nonenzymatic browning, and so on. [285].

*Orexigenic (Sanskr. Rocana).* Sour taste increases the appetite according to Ayurveda. Nevertheless, there are contradictory opinions in modern medicine concerning the orexigenic action of various sour items. Scientific studies on vinegar showed an opposite effect: vinegar (acetic acid) consumption reduced the voluntary food intake by increasing satiation during meals and decreasing appetite during subsequent meals [286]. Although, carbonated beverages induced secretion of hunger hormone ghrelin and increased food consumption in male rats [287]. Similar results on the levels of ghrelin hormone were found in 20 healthy human males upon drinking carbonated beverages [287].

*Increased Digestion and Metabolism (Sanskr. Agni Dipana)*. Kir2.1 K+ channel, known to be involved in sour taste transduction [185], is also expressed in parietal cells of the stomach, and it was suggested to be involved in controlling gastric acid secretion [26]. Short chain fatty acid acetate (a sour tastant) stimulates mitochondrial biogenesis via GPCR43 in brown adipocytes [288]. Brown adipocytes play an essential role in thermogenesis, as well as in the “burning” process of the excessive fats stored in the white adipose tissue [289].

*Beneficial for Heart (Sanskr. Hridya).* Ayurveda claims that sour taste is beneficial for heart [109, 110]. The most common compound having sour taste in our diet is ascorbic acid, a well-known antioxidant. Plasma level of vitamin C is a validated biomarker for fruit and vegetable intake, which reflect recent dietary intake of vitamin C [290]. A 20 *μ*mol/L increase in plasma vitamin C concentration (1 standard deviation) was associated with a 13% relative reduction in risk of atrial fibrillation in women, but there was no such association in men [291]. In another cohort study, even a small increase of 2 *μ*mol/L in plasma vitamin C was also associated with small reductions in anthropometric and metabolic cardiovascular risk factors [292]. Inverse association between plasma vitamin C and the risk of heart failure in the healthy population was found in a prospective study [293].

Sour taste is also the aspect of flavor most commonly associated with the short chain fatty acids (e.g., acetic acid, propionic acid, and butyric acid). A recent study showed that there is a mitochondrial preference for short chain fatty acid oxidation during coronary artery constriction [294]. All these studies suggest that sour tastants might play a cardioprotective role, but this issue needs to be validated by further studies.

Concerning the sour taste transducers, TASK-1 (one of the main K_2_P channels claimed to mediate sour taste in humans) [295], might have a functional importance in controlling the atrial size, repolarization of the cardiac action potential, maintenance of heart rate variability, and cardiac conduction system activity and has been proposed as a potential pharmacological target in case of atrial fibrillation [296–298].

Interestingly, Andersen-Tawil syndrome, also known as long QT-syndrome 7, is a rare autosomal dominant genetic disorder produced by mutations in gene codifying the potassium channel subunit Kir2.1 (an important component of the sour taste transducing pathway). This syndrome causes various life threatening cardiac disorders (ventricular arrhythmias, dilated cardiomyopathy) [299, 300]. 

*Excessive Sour Taste*. There is a trend all over the globe, to introduce more fermented food in the diet, due to their health benefits (e.g., probiotic component) [284].

According to a recent report, the global demand on dressing vinegar is also continually increasing: more than 115,000 metric tons were sold globally in 2016, while by the end of 2024, global consumption is predicted to reach 165,977 metric tons [301].

Globalisation of food requires frequent use of foods acidulants, for the previously mentioned purposes [285]. Although the demand of carbonated drinks like soda had an accelerated falling in the last 5 years, the producers supply, which contains more healthier carbonated beverages, is expected to capture consumer interest in the future (https://www.ibisworld.com/industry/default.aspx?indid=285). These data suggest that individuals would be more and more exposed to a kind of a “hidden” sour taste, as long as more fermented, acidulated, carbonated, and alcoholic dietary items are consumed.

One case-control clinical study has shown that excessive sour tasting food articles (mango, tomato, lime, citrus fruits, butter milk, tamarind, curd, and fermented items) produced dentine hypersensitivity, stomatitis, halitosis, heartburn, urticaria, papules, and joint inflammation [302], confirming the classical ayurvedic predictions [109]. Excessive use of sour taste causes burning sensation in the chest and cardiac region, according to Ayurveda [109], but it is not specified whether the pain has a cardiac or a digestive origin (cardiac ischemia or gastroesophageal acidic reflux).

Several surveys showed a weak association between carbonated beverages and tooth erosion and gastroesophageal reflux disease (if the consumption is more than 300 mL of a carbonated fluid) [303]. These type of drinks decreased ex vivo the microhardness of enamel [304]. Regarding Ayurveda, tooth sensitivity and burning sensation in throat, chest, or cardiac region may arise from excessive sourness [109, 110]. Soda, one of the carbonated beverage, as well as vinegar, evoke a burning sensation along with sour. This burning sensation is a kind of painful sensation mediated by CO_2_ via TRPA1 [146].

Another study found increased concentration of histamine and other biogenic amines (e.g., tyramine, putrescine, and cadaverine) in fermented food items (fermented sausage, fermented cheese, and smoked and salted fermented fish) [305]. Histamine in fermented food might produce itching and skin eruptions. These signs are also signs of “excessive ayur-sour taste” (Astanga Hridaya X.11) [110, 284].

If used in excess in isolation, sour taste might causes* mamsa vidaha*, a kind a muscle inflammation (*mamsa*: muscle,* vidaha*: turning acid, inflammation, burning) (Caraka Samhita, Sutrasthana XXVI.43). The alcoholic myositis of chronic drinkers is well known [306].

*Limitations of the Present Hypothesis on Extraoral Taste Receptors as Molecular Basis for Sour Taste (Amla Rasa) Ethnopharmacological Activities*. Sour taste is transduced by an intracellular acidification in taste bud cells, as previously mentioned [185]. Although sour tasting compounds might play the beneficial functions mentioned in Ayurveda (e.g., cardioprotective, apperitive, etc.), the direct contribution of sour taste signaling pathway to these effects is not yet entirely investigated. Moreover, other nontaste mechanisms might be involved (e.g., antioxidant activity of vitamin C).

### 6.5. Pungency (Sankr.* Katu Rasa*)

*Helps Intestinal Peristalsis.* According to Ayurveda, pungent remedies cure “intestinal torpor” or “intestinal inertia” (Sanskr.* alasaka*) (Astanga Hridaya. Sutrasthana X.17) [110, 307]. Aryl isothiocyanate and cinnamaldehyde, two pungent compounds that are TRPA1 agonists, improved intestinal transit in an animal model of postoperative ileus [144]. 6-Gingerol is another example of pungent compound TRPA1 agonist [142]. 6-Gingerol acted on the capsaicin-sensitive cholinergic neurons and modulated the contraction in isolated guinea pig ileum: lower concentrations enhanced capsaicin-induced contraction, while higher concentration inhibited them [308]. Capsaicin also stimulated motility of gastric antrum, duodenum, proximal jejunum, and proximal colon, when intragastrically administered in conscious dogs [309].

*Antiadiposity (Sanskr. Lekhanya). *Pungent remedies cure obesity, according to Ayurveda [109]. On the other side, beneficial effects of pungency transducer activation on adipose tissue were also reported in scientific studies. Capsaicin, a TRPV1 agonist, increased synthesis of hormone sensitive lipase, lipolysis, and reduced intracellular lipid content of adipocytes in vitro [310]. Dietary capsaicin decreased body weight, adipose tissue, obesity-induced insulin resistance, and hepatic steatosis in obese animals fed a high fat diet [311].

*Channels Dilatory (Sanskr. Srotamsi Vivrinoti)*. Ayurveda states that pungency dilates and opens the channels. According to modern science, a TRP-mediated vasodilation is induced by various dietary pungent agonists (e.g., carvacrol from oregano–TRPV3 agonist, eugenol from cloves–TRPV4 agonist), as well as by heat, and several mechanisms are involved (e.g., decrease in the intracellular Ca^2+^ of arterial myocytes, stimulation of nitric oxide, prostaglandin I2–PGI2, and endothelium-derived hyperpolarizing factor, EDHF production in endothelial vascular cells) [59, 131, 312–314]. 

*Anti-Infectious (Sanskr. Krimighna)*. In Ayurveda pungent remedies have anti-infectious properties [109, 110]. We have also performed a statistical analysis in a database containing 460 Indian medicinal plants mentioned in Ayurvedic Materia Medica and found a significant association between herbal pungency* (katu rasa)* and traditionally assigned anti-infectious activity* (krimighna)* (*p* < 0.001, OR = 3,43–8,17).

Today, scientists consider that pungency chemosensors, like TRPV1 and TRPA1, play protective roles in response to infectious stimuli [315]. Both TRPs are coexpressed in leukocytes and keratinocytes, cells with crucial functions in the systemic and local immune response [1, 315]. Activation of TRPV1 by capsaicin is associated with the release of two proinflammatory neuropeptides, substance P (SP) and calcitonin gene related peptide (CGRP), which may increase recruitment of leukocytes to the tissue and help in protection against sepsis [316]. TRPV1 knockout mice showed higher susceptibility to sepsis compared with wild type, due to several immune deficiencies related to macrophages (e.g., reduced phagocytosis, decreased reactive oxygen species, decreased bacteria clearance, and downregulation of tumour necrosis factor *α* expression) [317, 318]. In an animal model, cinnamaldehyde, a pungent compound which is also a TRPA1 agonist, reduced the severity of LPS-induced systemic inflammatory response syndrome through TRPA1-dependent but also TRPA1-independent mechanisms [319].

*Anti-Itching (Sanskr. Kandughna)*. Pungent remedies cure itching and allergy, according to Ayurveda [109, 110]. Science has already discovered that pungency chemosensors mediate or control itching. For instance, TRPV4 expressed in skin keratinocytes functions as a pruriceptor, mediating the histaminergic itch and scratching behavior [320]. TRPV4 antagonists attenuated serotonin-evoked scratching in vivo in animals [321]. TRPA1, TRPV4, and TRPV3 expressions were increased in burn scars with postburn pruritus [101]. Although, in terms of pharmacological activity, science differentiates between cooling and heating pungent compounds: cooling pungent compounds (TRPM8 agonists, e.g., menthol) ameliorate pruritus [322], while heating pungent compounds (TRPV1 agonists, capsaicin) aggravates it [323]. Scientists had recently suggested that agonists of cold transduction receptors might have antipruritic potential [322].

*Limitations of the Present Hypothesis on Extraoral TRPs as Molecular Basis for Pungency (Katu Rasa) Ethnopharmacological Activities*. Contrary to Ayurveda, pungency is not recognized as a taste in modern science. Although pungent compounds might play the beneficial functions mentioned in Ayurveda (e.g., anti-infectious, antiadiposity), the direct contribution of pungency chemosensorial transduction pathway to these effects remains to be largely determined.

### 6.6. Astringency (Sanskr.* Kashaya Rasa*)

*Antidiabetogenic (Sanskr. Medohara).* Astringent medicinal plants are more likely to have antidiabetogenic activity, according to traditional Ayurveda texts [109] and our previous statistical study on Ayurveda literature [159]. Proanthocyanidins, a category of condensed tannins, have shown activities that alleviate diabetes and its complications (e.g., neuropathy, retinopathy, and nephropathy), including hypoglycemic effect, and decreased concentration of advanced glycation end products [324]. 

*Healing of Wounds (Sanskr. Vranaropana).* Herbal astringency is correlated with the property of healing the wounds in Ayurveda [109, 110]. Scientific studies showed also the benefits of tannins in the management of skin and gastric ulcers. In animal experiments, the wound covered by a chitosan-gelatin sponge loaded with tannins and platelet-rich plasma healed quickly [325]. 

*Antihemorrhagic (Sanskr. Sonitasthapana).* Astringent medicinal plants and food alleviate bleeding disorders (Sanskr.* raktapitta*) [109], having antihemorrhagic activity. Tannins accelerate blood clotting [326]. Injection therapy with aluminum potassium sulfate and tannic acid (ALTA) produces a quick hemostatic effect in patients with internal hemorrhoids [327]. TAPE, a potent surgical biodegradable hemostatic glue based on tannic acid, was developed to be used efficiently against tissue bleeding [328]. 

*Antiadiposity (Sanskr. Medoshoshana)*. According to Ayurveda, astringency “dries the fat” [109, 110]. Modern science says that tannins decrease the serum lipid level [326]. For instance, dietary procyanidins reduced triglyceridemia in vivo in one animal model [329].

*Limitations of the Present Hypothesis on Extraoral Chemosensors as Molecular Basis for Astringency (Kashaya Rasa) Ethnopharmacological Activities*. In contrast with Ayurveda, modern science does not recognize astringency as a taste. Presently, there are a lot of controversially discussions on the possible mechanisms for astringency detection. This gap of knowledge hindered the development of the present hypothesis. Astringent compounds might play some of the pharmacological roles mentioned in Ayurveda (e.g., antihemorrhagic, vulnerary), but we found no scientific evidence for others (e.g., sedative, styptic). Also, the direct contribution of astringency chemosensorial transduction mechanism to these therapeutic activities remains obscure.

## 7. Postdigestive Effect (*Vipaka*) or the Taste Beyond the Taste

Every food item consisting of certain proportions of different nutrients (e.g., proteins, lipids, carbohydrates, vitamins, and minerals) undergoes biochemical changes during digestion and metabolism. Similarly, Ayurveda claims that every food item has a unique composition of five elements or* pancha mahabhutas* (earth:* prithivi*, water:* apas*, fire:* tejas*, air:* vayu*, and ether:* akasha*). The nutrient may retain or not its elemental composition throughout the digestion and metabolism. The postdigestive elemental configuration is evaluated in Ayurveda through the concept of* vipaka* (postdigestive effect). Therefore* vipaka* is “the taste after the substance is digested” (Caraka Samhita, Sutrasthana XXVI.63/Ayurveda Dipika) [109]. Here, the term “taste” should be understood within the Ayurveda framework (as combination of two elements, e.g., sweet,* prithivi* and* apas*), not as sensorial property of the nutrient. Similarly with taste receptors, which have taste roles and extrataste roles, nutrients have sensorial taste properties* (rasa)* and extrasensorial taste properties* (vipaka)*. Some of the nutrients are chemically modified by the time they reach the extraoral taste receptors in the intestine (in ayurvedic language they come to have a different composition of* pancha mahabhutas *from the original nutrient). The resultant digestion products have a “latent taste” attribute, which does not induce a sensorial experience, but display a specific affinity for a certain type of extraoral taste receptors. For instance, dietary sucrose (having a sweet taste, or sweet* rasa*), is converted by intestinal sucrase into glucose and fructose, which have also a sweet “latent taste” (sweet* vipaka*) and therefore affinity for the sweet extraoral taste receptors.

Although these extraoral taste receptors do not mediate any taste sensation, they are still called “taste receptors.” Similarly, although the products of digestion do not mediate any taste sensation, they are still characterized in terms of* rasa* (taste), and the molecular basis for this* rasa* characteristic might be their potential affinity for the extraoral taste receptors.

All nutrients show mainly three types of* vipaka*, which depends on their* rasa* (tastes) (Table 2) [109, 330]. Since* vipaka* is expressed in taste modalities (sweet, sour, and pungent) at both digestive and postdigestive levels (sweet* vipaka* helps in elimination of stool and urine and increases semen (*shukra*); pungent* vipaka* obstructs the elimination of stool and urine and decreases semen; sour* vipaka *helps in elimination of stool and urine and reduces semen) [109], it would be reasonable to hypothesize that* vipaka* is mediated through extraoral taste receptors or TRPs. In our view,* vipaka* is a much more complex concept and difficult to be understood than* rasa *(many related aspects cannot be explained, e.g., why the bitter and astringent tasting compounds have a pungent* vipaka*), but we estimate that its significance will be gradually deciphered, as the physiological functions of extraoral taste receptors and TRP channels will be revealed and more taste assessment studies on medicinal plants and phytochemicals will be performed.

## 8. Energetic Nature (*Virya*) Mediated by TRPs

Herbal qualities* (gunas)* (e.g., dry-wet, light-heavy) represent other ethnopharmacological descriptors used in Ayurveda [331]. For instance, hot-cold properties, also known as* virya* or potency of the herb, are considered traditionally to be responsible for various activities. Medicinal plants with “hot” potency* (ushna virya)* produce a sensation of heat, have drying effect, and help digestion. Black pepper, ginger, and cayenne are typical hot medicinal plants. Medicinal plants with “cold” potency* (shita virya)* induce a cooling sensation and are pacifying and nourishing. Licorice, sandalwood, aloe, and arjuna are examples of cold medicinal plants [109, 119].

This pair of qualities (hot-cold) is used for millennia, in conjunction with herbal taste* (rasa)* to select the most appropriate medicinal plants for a patient in Ayurveda. Hot-cold classification of herbal remedies is quite common in many medical cultures all over the world (China, Europe, Mesoamerica, South America, Iran, etc.) [332–335]. Although certain critics considered hot-cold based selection approach of herbals to be inconsistent or abstract [333, 336], recent scientific arguments support it. A close relationship between families/major chemical compounds of medicinal plants and their hot-cold qualities was noticed [337, 338], although a previous study denied it [333]. Several teams of scientists reported its potential physiological basis, by showing that hot-cold medicinal plants may influence differently various parameters: the capillary red blood cell velocity in nail fold microcirculation, pulse characteristics on sphygmogram, hormonal concentrations (serum thyroxin, triiodothyronine, corticosterone, and urine vanillylmandelic acid), metabolic parameters (serum glucose, triglycerides), body weight, enzymatic activities (liver succinate dehydrogenase, liver Na^+^-K^+^-ATPase), muscle glycogen, and so on. [334, 339–342].

An excellent experimental study performed in Zoque community of healers (Mexico) has even showed that these humoral qualities represent the key predictor of herbal therapeutic uses, not being only a post hoc attribution [208]. Scientists concluded that hot-cold properties represent “an important cultural filter connecting organoleptic properties and therapeutic uses” of medicinal plants [208]. In terms of molecular basis of this nexus, we estimate that certain categories of chemosensors (e.g., TRP) may be involved. For instance, it seems that TRPs are involved not only in pungency sensation, but also in thermosensation transduction. When activated by phytochemicals, TRPs stimulate either a sensation of warmth or a sensation of cooling: TRPV1, TRPV3, and TRPV4 channels are involved in heat sensation, such as that induced by chili peppers (capsaicin) or ginger, while TRPM8 channels are associated with cold sensation, such as that induced by mint (Table 4) [1]. Interestingly, taste-guided identification of natural TRPs agonists from plants has been suggested as a bioprospecting method that might be used for development of new pharmaceutical agents [148]. 

*Cold Virya and Trigeminal Chemosensitivity.* The trigeminal nerve responsible for the somatic innervation in the oral mucosa contains numerous transient receptor potential melastatine 8 (TRPM8) channels, which are receptive to cold stimulus [1, 343]. Several typical phytochemicals derived from plants known to produce a cooling sensation are TRPM8 agonists: menthol [123], geraniol [125], and eucalyptol [125] (Table 4). 

*Cold Virya and Taste Cells*. TRPM5 channel, which is expressed in T1R and T2R families of taste cells (and not expressed in sensory neurons), is recognized as a taste-specific channel common to sweet, umami, and bitter-responding cells, but not salt and sour-responding cells [135, 158]. TRPM5 is activated downstream of the G protein-coupled taste receptors [158]. TRPM5 is also thermally sensitive, its activity increasing from 15 to 35°C [121]; therefore it is responsible for the taste transduction triggered by cold stimulus. Thus, scientists concluded that cold stimulus should selectively influence the perception of sweet, umami, and bitter [344]. These facts are interesting from an ayurvedic point of view, since sweet and bitter tastes are considered “cold”* (shita virya)* in Ayurveda, while salty and sour are regarded as “hot”* (ushna virya)*. Might TRPM5 mediate, more or less, the cold* virya* of sweet and bitter medicinal plants? Further experimental studies are required to answer this question. 

*Hot Virya, Taste Cells, and Trigeminal Chemosensitivity*. TRPV1 expression in taste cells is debatable. TRPV1 transcripts have been identified in the RNA extracted from taste buds, but contamination from trigeminal endings or extragustative oral epithelium, which also expressed TRPV1, could not be ruled out [1, 345]. TRPV1 is activated by pungent compounds (capsaicin, piperine), salty stimuli, low pH, and noxious heat (Table 4) [135–138, 346]. These facts are significant from an ayurvedic point of view, since pungency and salty taste are considered “hot”* (ushna virya)*.

TRPV3 and TRPV4 are also expressed in the trigeminal nerve and extragustative oral epithelium, being activated by heat and various pungent compounds. Might TRPV1, V3, and V4 mediate, more or less, the “hot” quality of salty and pungent remedies?

Further experimental studies are required in order to understand the contribution of TRPs to the multimodal experience of* rasa.*

Mammalian TRP channels have not only oral location, but a broad tissue distribution (at least one type of TRP present virtually in all cells), this ubiquity bringing them to the vanguards of the chemosensorial system [132, 347, 348].

Since the effects of* virya* are local (oral), as well as general (extraoral), it would be reasonable to claim that the systemic/extraoral effects of* virya* may be mediated, at least in part, by extraoral chemosensorial transducers (e.g., TRP), which may (e.g., TRPM5) or may not (e.g., TRPM8) be part of the taste signaling pathways.

Nevertheless, this hypothesis on TRPs as mediator of* virya* has several limitations, not offering explanations for certain somewhat puzzling facts. For instance, why some potent agonists of TRPV1 (which is a capsaicin-like pungency transduction channel) lack irritancy or do not induce a painful hot sensation (ayur-*“hot virya”*) when applied to the mucosal surfaces [349]?

Why eugenol, the main phytochemical responsible for clove aroma, was found to have hypothermic effects [120], while clove is described as being “hot” in Ayurveda [338]? Why eugenol induces hypothermic effects [120], although it activates TRPV3 and TRPV1 (warmth transducers) [349]? One potential explanation lies in the ligand promiscuity of eugenol, which activates several TRPs, including TRPA1 [349]. TRPA1 activation elicits dual sensations, either a pungency, or a painful sensation, thus offering a potential molecular model explaining why noxious cold can paradoxically be perceived as a burning sensation [128].

## 9. Limitations of Taste as (ethno)pharmacological Descriptor

The taste as an (ethno)pharmacological descriptor has several limitations. Is taste only a cultural filter for selection of herbs or does it have a physiological/biological basis? The answer to this question is beyond the purpose of the present paper, and it may be even unaccomplishable. Experts suggested that “Unfortunately, it is not possible to deduce the origin of plant uses in ethnobotanical research” and therefore we could have only partial answers concerning the reasons beyond the associations between medicinal plants and their traditional indications [210]. To our knowledge, there are no experimental studies that could prove a causal relationship between the herbal taste and the therapeutic activities.

Another limitation of this potential (ethno)pharmacological descriptor is represented by the extreme inter- and intravariability of taste perception. Genetic, physiological, and hormonal variation causes differences in bitter taste perception [152, 350–352]. Intraindividual sensitivity to a particular taste may vary considerably from one moment to another, being influenced by diverse factors such as smoking, fatigue, alcohol intake, and ingestion of strongly flavored food [353, 354]. Taste preferences, interpretation, and evaluation are to a large degree determined by culture, and they may further be learnt through cultural familiarity with certain foods, herbs, or drinks [355, 356], varying also between males and females [357]. Lastly, tastes are extremely variable as they may be masked either by certain competitive agonists for TR, or by other tastes [355, 358].

Another important limitation is derived from the existence of the extrataste effects of tastants, which may also have other pharmacological targets than TR, such as enzymes, ion channels, transport proteins, or other receptors [359]. Ayurveda, as well, mentions that* rasa* has not absolute supremacy in terms of therapeutic activity (e.g., certain plants act through their* virya*-hot/cold qualities, others through* vipaka*–postdigestive effect or* prabhava*–special potency). As a rule,* virya* and* vipaka* may be inferred from* rasa*, and we estimated that they are at least partially mediated by (extra) oral taste receptors or chemosensorial transducers. Although,* prabhava* is not inferred from* rasa*; therefore it may be related to these extrataste pharmacological activities.

## 10. Limitations of the Present Hypothesis on Extraoral Taste Receptors as Molecular Basis for the Biological Functions of* Rasa* (Ayur-Taste)

The majority of the published studies on extraoral taste receptors did not show that tastant effects are mediated by taste receptors, being based on observations made in parallel and are thus not causally linked. Knockout mouse studies are recommended to support the causal relationship and scientists expect that these will facilitate our understanding on the functional roles of extraoral taste receptors [269].

There are functional differences among mice and human taste receptor orthologs [157], and this requires adjustment of wrong assumptions that there is an identity of receptorial pharmacological profile between various species. For instance, different bitter agonists activate homologous bitter receptors in mice and man [157]. It is not known yet whether mice and man might have specialized TR for compounds of species-specific relevance [157].

Unfortunately, the scarcity of available data on the functional properties of extraoral taste receptors does not provide yet a full insight into the molecular basis of ayur-tastes and other herbal attributes* (vipaka, virya)*.

## 11. Concluding Remarks and Future Directions

This recently discovered* diffuse chemosensory system* based on extraoral taste cells represents a potential new drug target [196], and its manipulation through tastants could be a new frontier in both modern and traditional pharmacology.

Many aspects of the basic tastes and trigeminal orosensations are yet to be understood. Although progress has been done in discovering the functional roles of extraoral taste receptors, these are still largely not known. Further experimental and clinical studies should investigate whether a particular taste could trigger certain pharmacological effects.

The tastants activate the taste receptors, but there are also chemicals, including phytochemicals, which, on the contrary, block the taste receptors (e.g., 6-methoxyflavanones – blockers for human bitter taste receptor T2R39) [360]. Up to date, these so called receptor blockers are mainly studied or used to mask certain unpleasant tastes, such as bitterness of drugs, food, or beverages [270, 358, 360, 361], but beyond such effect, these antagonists may also play other biological roles, mediated by their binding to extraoral taste receptors. Further studies are required to understand the interplay between these opposite ligands (agonists versus antagonists), their interaction with extraoral taste receptors, and their potential therapeutic roles.

Complete mapping of human body in terms of taste receptor and TRP expression is highly required in order to reveal the full meaning of* rasa, vipaka,* and* virya *concepts and their significance for health. More experimental studies that investigate comparative tissue distribution of taste receptors in the body, such as those already performed [27, 60, 362, 363], are crucial for understanding the role of taste in health and disease.

The discovery of several polymorphisms in the taste receptor genes has also raised questions regarding the potential role of these genetic variations in individual predisposition to certain diseases [150]. It would be reasonable to state that the constitutional types described in Ayurveda* (Vata, Pitta, and Kapha)* based on phenotype features, which are traditionally considered to be prone to develop certain specific disorders, may also be characterized by different polymorphisms in the taste receptor or TRP genes.

Integrative research in the field of ethnomedicine can do more than only speed up bioprospection for development of new drugs. It can lead to paradigm shift in all areas of medicine, revealing innovative treatments, enriching our understanding about the healing process, and opening new unexpected perspectives for human wellbeing improvement.

However, the integrative research in the field of ethnopharmacology and ethnomedicine is still in its infancy, and more efforts are required in order to reveal the entire ethnomedical significance of taste and of other ethnopharmacophacological descriptors. This scientific endeavor is not only necessary, but also urgent, in order to prevent an irreversible traditional knowledge loss. Concerning the methodology that should be used in ethnopharmacological research, Reyes-Garcia suggested that “the efficacy of a medicinal plant should be measured in a culturally appropriated way, and the failure to consider the cultural context within which plants are used can result in misunderstandings of a plant's efficacy.” [364].

## Figures and Tables

**Table 1:** Expression of taste receptor elements and other chemosensorial transducers in various organs or tissues (BAT: brown adipose tissue, H: evidence of TR expression in human tissues, and ?: not yet studied).

Organ	Sweet (T1R)	Bitter (T2R)	Sour (T_2_P)	Salty (ENaC)	Pungent (TRP)
Stomach	(+) [22]	(+H) [23, 24]	(+H) [25, 26]	(+H) [27]	(+) [28]
Small intestine	(+) [29]	(+) [24]	(+H) [25]	(+) [30]	(+H) [31]
Colon	(+) [23]	(+H) [32]	(+H) [33]	(+) [34]	(+H) [35]
Pancreas	(+) [36]	(+H) [37]	(+H) [25, 33]	(+H) [38, 39]	(+H) [40]
Spleen	(+H) [41, 42]	(+H) [41, 42]	(+H) [25]	(+H) [27]	(+) [43]
Liver	+ bile ducts (−) hepatocytes [44]	(+H) [41, 42]	(+H) [25]	(+H) [27, 39]	(+) [43]
Kidney	(+) [45]	(+H) [41, 42, 46]	(+H) [25, 47]	(+H) [39]	(+) [48]
Urinary bladder	(+) [49]	(+H) [41, 42]	(+H) [50]	(+H) [51]	(+H) [52]
Heart	(+) [20]	(+) [53]	(+) [33, 54, 55]	(+H) [27]	(+) [56]
Vessels	?	(+) [57]	(+) [54]	(+) [58]	(+H) [59]
Brain	(+) [16]	(+) [60]	(+H) [54, 61, 62]	(+H) [38, 63]	(+) [43]
Respiratory system	(+) [64]	(+) [65]	(+H) [25]	(+H) [39]	(+H) [66]
Adipose tissue	(+) [21]	(+) [67]	(+BAT) [68]	?	(+) [43]
Bone	?	(+) osteoclasts [69]	(+H) [70, 71]	(+H) [72, 73]	(+H) [74]
Bone marrow	?	(+) [75]	(+H) [76]	(+H) [27]	(+) [77]
Joints	?	?		(+H) [78]	(+) [79]
Leukocytes	(+H) [80, 81]	(+) [82]	(+) [83]	(+H) [27]	(+H) [84]
Testis, sperm	(+) [85]	(+) [86]	(+) [87]	(+H) [27, 38]	(+) [43, 88]
Ovary	?	(+) [60]	(+) [89]	(+H) [38]	(+) [90, 91]
Uterus	?	(+H) [41, 42]	(+H) [25]	(+) [92]	(+) [93]
Breast	(+H) [41, 42]	(+) [94]	(+) [95]	(+H) [96]	(+) [97]
Skin	?	(+H) [98]	(+) [99]	(+) [100]	(+H) [101, 102]
Thyroid	?	(+) [103]	(+H) [104]	(+) [105]	?
Thymus	(+H) [81]	(+) [60]	(+H) [106]	(+H) [27]	?
Adrenal glands	(+) [107]	(+H) [41, 42]	(+) [108]	(+H) [27]	?
Pituitary gland	(+H) [41, 42]	(+H) [41, 42]	(+H) [25]	?	?

**Table 2:** Ethnopharmacological description of the six tastes *(rasa)* in Ayurveda [109, 110].

Rasa (taste)	Virya (energetic nature)	Guna (qualities)	Vipaka (postdigestive effect)	Karman (therapeutic activities)
Sweet	Cold	Heavy, wet	Sweet	Nourishing of plasma, blood, muscle, adipose tissue, bone, marrow, and semen, longevity enhancer, strengthening, antitoxic, antidipsogenic, sensorial soothing

Salty	Hot	Heavy, wet	Sweet	Carminative, digestive, laxative, deobstruent, lubrifying, sialogogue

Sour	Hot	Light, wet	Sour	Digestive stimulant, nourishing the heart and the entire body, sialogogue, stimulates appetite, emollient, strengthening the sense organs

Pungent	Hot	Light, dry	Pungent	Digestive stimulant, anti-itching, anti-infectious, reduces the muscle mass, breaks obstructions (e.g., anticoagulant), purifying, clarifies the passages, helps sensorial activity, intestinal peristalsis, elimination of waste products (e.g., feces), antiswelling, channel dilatory

Bitter	Cold	Light, dry	Pungent	Antianorexia, digestive stimulant, antitoxic, anti-infectious, febrifuge, antiemetic, milk purification, reducer of adipose tissue, muscle fat, bone marrow, lymph, pus, sweat, urine, stool

Astringent	Cold	Heavy, dry	Pungent	Sedative, styptic, constipative (antidiarrheic), antihemorrhagic, absorptive, blood purifier, skin purifier, wound/ulcer healing, anti-adiposity

**Table 3:** Comparative effects of excessive dietary saltiness in Ayurveda and modern medicine (*∗* = not yet clarified/studied, *∗∗* = contradictory information Ayurveda versus modern science).

Effect of excessive use of drugs and diet having salty taste, according to Ayurveda [109]	High salt diet effects according to scientific studies
Bursting of inflamed part, heating sensation, inflammatory skin diseases (e.g., *visarpa – *erysipelas, herpes*, vicharcika–*psoriasis^*∗*^)	Proinflammatory effect via induction of pathogenic Th17 cells and cytokine synthesis [111, 112], increased risk of inflammatory bowel diseases [113], reduced immune tolerance and increased risk of autoimmune diseases [112]

Aggravation of Blood *(rakta)*, bleeding from different parts of the body	Hypertension, epistaxis, stroke, intracerebral hemorrhage [114, 115]

Obstruction of the function of senses, fainting, depletion of muscles	Multiple sclerosis (double vision, blindness, muscle weakness, sensorial and coordination disorders) (>5 g NaCl/day) [111]

Gastric hyperacidity *(amlapitta)* ^*∗∗*^	High level of gastric inflammation [116], but paradoxical protection against duodenal ulcers by decreasing acid output^*∗∗*^

Premature skin ageing (wrinkling, graying, baldness)^*∗*^	Potentially linked with excessive storage of Na^+^ in skin without concomitant water retention, leading to an osmotic stress in the skin microenvironment [117], increased oxidative stress and accumulation of peroxidative damages [118]

**Table 4:** Transducers involved in thermosensation and examples of agonists (^*∗*^contradictory information Ayurveda versus modern science: evergreen tree and *Andrographis paniculata* are described as being “cold” in Ayurveda [119] and clove is described as being “hot” in Ayurveda [119], while eugenol was found to have hypothermic effects [120]; mint is pungent and therefore “hot” although it induces a cooling sensation [119]).

TRP channels	Agonists
Cooling Sensation	

TRPM5 (sweet, umami, bitter taste receptor cells) (15°–35°C) [121]	Rutamarin (rue) [122]

TRPM8 (trigeminal orosensation) Moderate non-painful coolness (10°–28°C) [123, 124]	Menthol (mint)^*∗*^ [123] Geraniol (rose oil, citronella oil) [125] Eucalyptol (eucalyptus) [125]

TRPA1 (trigeminal orosensation) Noxious cold or painful cold (<15°C) [126, 127]	Eugenol (clove) [128] See also TRPA1 (pungency) under Warming sensation

Warming sensation	

TRPV4 (trigeminal orosensation) (>27°C) non painful warmth [129]	Bisandrographolide A *(Andrographis paniculata)* ^*∗*^ [130] Eugenol (clove)^*∗*^ [131]

TRPV3 (trigeminal orosensation) (≥33°C) non painful warmth [132]	Carvacrol (oregano) [133] Eugenol (clove)^*∗*^ [133] Thymol (thyme) [133] Camphor (evergreen tree)^*∗*^ [134]

TRPV1 (trigeminal orosensation) (>43°C) painful heat [135], pungency	Capsaicin (chili peppers) [136] Piperine (black pepper) [137] Low pH (<5.9) [138] (sour items?)

TRPV2 (trigeminal orosensation) (>52°C) extreme heat [135]	Δ9-Tetrahydrocannabinol, Δ9-tetrahydrocannabinol acid [139], cannabidiol (marijuana) [140]

TRPA1 (trigeminal orosensation – pungency, burning sensation)	Allicin (garlic) [141] 6-Gingerol, zingerone, 6-shogaol (ginger) [142] Allyl isothiocyanate (horseradish, mustard oil) [143] Cinnamaldehyde (cinnamon) [144] Oleocanthal (olive oil) [145] Organic weak acids [146] (sour plants?) Polygodial (water pepper) [147] Perillaketone, perillaldehyde *(Perilla frutescens)* [148]

## Abbreviations

CD36: Cluster of differentiation 36
ENaC: Epithelial sodium channels
FFA: Free fatty acids
GPCR: G protein-coupled receptors
K_2_P: 2-pore-domain potassium channel
LCFA: Long chain fatty acids
PKDL: Polycystic kidney disease protein-like
sanskr.: Sanskrit
TR: Taste receptor
T1R2/T1R3: Sweet taste receptor
T1R1/T1R3: Umami taste receptor
T2R: Bitter taste receptor
TRC: Taste receptor cell
TRP: Transient receptor potential channel
TRPM: Transient receptor potential melastatine.

## References

[1] Roper S. D. (2014). TRPs in Taste and Chemesthesis. *Handbook of Experimental Pharmacology*.

[2] Lindemann B. (1996). Taste reception. *Physiological Reviews*.

[3] Liman E. R., Zhang Y. V., Montell C. (2014). Peripheral coding of taste. *Neuron*.

[4] Huang Y. A., Maruyama Y., Stimac R., Roper S. D. (2008). Presynaptic (Type III) cells in mouse taste buds sense sour (acid) taste. *Journal of Physiology*.

[5] Bigiani A. (2001). Mouse taste cells with glialike membrane properties. *Journal of Neurophysiology*.

[6] Lewandowski B. C., Sukumaran S. K., Margolskee R. F., Bachmanov A. A. (2016). Amiloride-insensitive salt taste is mediated by two populations of type III taste cells with distinct transduction mechanisms. *Journal of Neuroscience*.

[7] Chandrashekar J., Kuhn C., Oka Y. (2010). The cells and peripheral representation of sodium taste in mice. *Nature*.

[8] Yoshida R., Horio N., Murata Y., Yasumatsu K., Shigemura N., Ninomiya Y. (2009). NaCl responsive taste cells in the mouse fungiform taste buds. *Neuroscience*.

[9] Roper S. D. (1989). The cell biology of vertebrate taste receptors. *Annual Review of Neuroscience*.

[10] Sullivan J. M., Borecki A. A., Oleskevich S. (2010). Stem and progenitor cell compartments within adult mouse taste buds. *European Journal of Neuroscience*.

[11] Zhao G. Q., Zhang Y., Hoon M. A. (2003). The receptors for mammalian sweet and umami taste. *Cell*.

[12] Schobel N., Radtke D., Kyereme J. (2014). Astringency is a trigeminal sensation that involves the activation of G protein-coupled signaling by phenolic compounds. *Chemical Senses*.

[13] Huang A. L., Chen X., Hoon M. A. (2006). The cells and logic for mammalian sour taste detection. *Nature*.

[14] Li F. (2013). Taste perception: from the tongue to the testis. *Molecular Human Reproduction*.

[15] Henquin J.-C. (2012). Do pancreatic *β* cells "taste" nutrients to secrete insulin?. *Science Signaling*.

[16] Ren X., Zhou L., Terwilliger R., Newton S. S., de Araujo I. E. (2009). Sweet taste signaling functions as a hypothalamic glucose sensor. *Frontiers in Integrative Neuroscience*.

[17] Raybould H. E. (1998). Does your gut taste? Sensory transduction in the gastrointestinal tract. *News in Physiological Sciences*.

[18] Dehkordi O., Rose J. E., Fatemi M. (2012). Neuronal expression of bitter taste receptors and downstream signaling molecules in the rat brainstem. *Brain Research*.

[19] Yamamoto K., Ishimaru Y. (2013). Oral and extra-oral taste perception. *Seminars in Cell and Developmental Biology*.

[20] Foster S. R., Porrello E. R., Purdue B. (2013). Expression, regulation and putative nutrient-sensing function of taste gpcrs in the heart. *PLoS ONE*.

[21] Masubuchi Y., Nakagawa Y., Ma J. (2013). A novel regulatory function of sweet taste-sensing receptor in adipogenic differentiation of 3T3-L1 cells. *PLoS ONE*.

[22] Hass N., Schwarzenbacher K., Breer H. (2010). T1R3 is expressed in brush cells and ghrelin-producing cells of murine stomach. *Cell and Tissue Research*.

[23] Rozengurt N., Wu S. V., Chen M. C., Huang C., Sternini C., Rozengurt E. (2006). Colocalization of the *α*-subunit of gustducin with PYY and GLP-1 in L cells of human colon. *The American Journal of Physiology—Gastrointestinal and Liver Physiology*.

[24] Wu S. V., Rozengurt N., Yang M., Young S. H., Sinnett-Smith J., Rozengurt E. (2002). Expression of bitter taste receptors of the T2R family in the gastrointestinal tract and enteroendocrine STC-1 cells. *Proceedings of the National Academy of Sciences*.

[25] Medhurst A. D., Rennie G., Chapman C. G. (2001). Distribution analysis of human two pore domain potassium channels in tissues of the central nervous system and periphery. *Brain Research Molecular Brain Research*.

[26] Malinowska D. H., Sherry A. M., Tewari K. P., Cuppoletti J. (2004). Gastric parietal cell secretory membrane contains PKA- and acid-activated Kir2.1 K+ channels. *American Journal of Physiology*.

[27] Yamamura H., Ugawa S., Ueda T., Nagao M., Shimada S. (2004). Protons activate the *δ*-subunit of the epithelial Na+ channel in humans. *Journal of Biological Chemistry*.

[28] Mihara H., Suzuki N., Boudaka A. A. (2016). Transient receptor potential vanilloid 4-dependent calcium influx and ATP release in mouse and rat gastric epithelia. *World Journal of Gastroenterology*.

[29] Dyer J., Salmon K. S. H., Zibrik L., Shirazi-Beechey S. P. (2005). Expression of sweet taste receptors of the T1R family in the intestinal tract and enteroendocrine cells. *Biochemical Society Transactions*.

[30] Koyama K., Sasaki I., Naito et al. H. (1999). nduction of epithelial Na+ channel in rat ileum after proctocolectomy. *American Journal of Physiology*.

[31] Yamawaki H., Mihara H., Suzuki N. (2014). Role of transient receptor potential vanilloid 4 activation in indomethacin-induced intestinal damage. *American Journal of Physiology - Gastrointestinal and Liver Physiology*.

[32] Kaji I., Karaki S.-I., Fukami Y., Terasaki M., Kuwahara A. (2009). Secretory effects of a luminal bitter tastant and expressions of bitter taste receptors, T2Rs, in the human and rat large intestine. *The American Journal of Physiology—Gastrointestinal and Liver Physiology*.

[33] Duprat F., Lesage F., Fink M., Reyes R., Heurteaux C., Lazdunski M. (1997). TASK, a human background K+ channel to sense external pH variations near physiological pH. *EMBO Journal*.

[34] Kunzelmann K., Mall M. (2002). Electrolyte transport in the mammalian colon: mechanisms and implications for disease. *Physiological Reviews*.

[35] D'Aldebert E., Cenac N., Rousset P. (2011). Transient receptor potential vanilloid 4 activated inflammatory signals by intestinal epithelial cells and colitis in mice. *Gastroenterology*.

[36] Nakagawa Y., Nagasawa M., Yamada S. (2009). Sweet taste receptor expressed in pancreatic beta-cells activates the calcium and cyclic AMP signaling systems and stimulates insulin secretion. *PLoS ONE*.

[37] Gaida M. M., Mayer C., Dapunt U. (2016). Expression of the bitter receptor T2R38 in pancreatic cancer: Localization in lipid droplets and activation by a bacteria-derived quorum-sensing molecule. *Oncotarget*.

[38] Waldmann R., Champigny G., Bassilana F., Voilley N., Lazdunski M. (1995). Molecular cloning and functional expression of a novel amiloride-sensitive Na+ channel. *Journal of Biological Chemistry*.

[39] McDonald F. J., Snyder P. M., McCray P. B. J., Welsh M. J. (June 1994). Cloning, expression, and tissue distribution of a human amiloride-sensitive Na+ channel. *American Journal of Physiology*.

[40] Fagelskiold A. J., Kannisto K., Bostrom et al. A. (2012). Insulin-secreting INS-1E cells express functional TRPV1 channels. *Islets*.

[41] Uhlen M., Fagerberg L., Hallstrom B. M. (2015). Tissue-based map of the human proteome. *Science*.

[42] http://www.proteinatlas.org/

[43] Nilius B., Vriens J., Prenen J., Droogmans G., Voets T. (2004). TRPV4 calcium entry channel: a paradigm for gating diversity. *The American Journal of Physiology—Cell Physiology*.

[44] Taniguchi K. (2004). Expression of the sweet receptor protein, T1R3, in the human liver and pancreas. *Journal of Veterinary Medical Science*.

[45] Kiuchi S., Yamada T., Kiyokawa N., Saito T., Fujimoto J., Yasue H. (2006). Genomic structure of swine taste receptor family 1 member 3, TAS1R3, and its expression in tissues. *Cytogenetic and Genome Research*.

[46] Liu X., Gu F., Jiang L., Chen F., Li F. (2015). Expression of bitter taste receptor Tas2r105 in mouse kidney. *Biochemical and Biophysical Research Communications*.

[47] Reyes R., Duprat F., Lesage F. (1998). Cloning and expression of a novel pH-sensitive two pore domain K+ channel from human kidney. *Journal of Biological Chemistry*.

[48] Wissenbach U., Bödding M., Freichel M., Flockerzi V. (2000). Trp12, a novel Trp related protein from kidney. *FEBS Letters*.

[49] Elliott R. A., Kapoor S., Tincello D. G. (2011). Expression and distribution of the sweet taste receptor isoforms T1R2 and T1R3 in human and rat bladders. *Journal of Urology*.

[50] Lei Q., Pan X.-Q., Chang S., Malkowicz S. B., Guzzo T. J., Malykhina A. P. (2014). Response of the human detrusor to stretch is regulated by TREK-1, a two-pore-domain (K2P) mechano-gated potassium channel. *Journal of Physiology*.

[51] Araki I., Du S., Kamiyama M. (2004). Overexpression of epithelial sodium channels in epithelium of human urinary bladder with outlet obstruction. *Urology*.

[52] Zhang H. Y., Chu J. F., Li P., Li N., Lv Z. H. (2015). Expression and diagnosis of transient receptor potential vanilloid1 in urothelium of patients with overactive bladder. *Journal of Biological Regulators and Homeostatic Agents*.

[53] Foster S. R., Blank K., See Hoe L. E. (2014). Bitter taste receptor agonists elicit G-protein-dependent negative inotropy in the murine heart. *FASEB Journal*.

[54] Kubo Y., Baldwin T. J., Nung Jan Y., Jan L. Y. (1993). Primary structure and functional expression of a mouse inward rectifier potassium channel. *Nature*.

[55] Matamoros M., Perez-Hernández M., Guerrero-Serna G. (2016). Nav1.5 N-terminal domain binding to *α*1-syntrophin increases membrane density of human Kir2.1, Kir2.2 and Nav1.5 channels. *Cardiovascular Research*.

[56] Randhawa P. K., Jaggi A. S. (2015). TRPV4 channels: physiological and pathological role in cardiovascular system. *Basic Research in Cardiology*.

[57] Chen J. G., Ping N. N., Liang et al. D. (2017). The expression of bitter taste receptors in mesenteric, cerebral and omental arteries. *Life Sciences*.

[58] Drummond H. A., Gebremedhin D., Harder D. R. (2004). Degenerin/epithelial Na^+^ channel proteins: components of a vascular mechanosensor. *Hypertension*.

[59] Pu J., Wang Z., Zhou et al. H. (2015). Role of TRPV4 channels in regulation of eNOS expression in brain microvascular endothelial cells under the condition of mechanical stretch. *Journal of Central South University*.

[60] Voigt A., Hubner S., Doring L. (2015). Cre-mediated recombination in Tas2r131 cells-A unique way to explore bitter taste receptor function inside and outside of the taste system. *Chemical Senses*.

[61] Rusznak Z., Pocsai K., Kovacs et al. I. (2004). Differential distribution of TASK-1, TASK-2 and TASK-3 immunoreactivities in the rat and human cerebellum. *Cellular and Molecular Life Sciences*.

[62] Nguyen H. M., Grossinger E. M., Horiuchi M. (2017). Differential Kv1.3, KCa3.1, and Kir2.1 expression in “classically” and “alternatively” activated microglia. *Glia*.

[63] Teruyama R., Sakuraba M., Wilson L. L., Wandrey N. E. J., Armstrong W. E. (2012). Epithelial Na^+^ sodium channels in magnocellular cells of the rat supraoptic and paraventricular nuclei. *American Journal of Physiology—Endocrinology and Metabolism*.

[64] Braun T., Mack B., Kramer M. F. (2011). Solitary chemosensory cells in the respiratory and vomeronasal epithelium of the human nose: a pilot study. *Rhinology*.

[65] Shah A. S., Yehuda B.-S., Moninger T. O., Kline J. N., Welsh M. J. (2009). Motile cilia of human airway epithelia are chemosensory. *Science*.

[66] Fernández-Fernández J. M., Nobles M., Currid A., Vázquez E., Valverde M. A. (2002). Maxi K^+^ channel mediates regulatory volume decrease response in a human bronchial epithelial cell line. *The American Journal of Physiology—Cell Physiology*.

[67] Ning X., He J., Shi X., Yang G. (2016). Regulation of adipogenesis by quinine through the ERK/S6 pathway. *International Journal of Molecular Sciences*.

[68] Pisani D. F., Beranger G. E., Corinus A. (2016). The K+ channel TASK1 modulates b-adrenergic response in brown adipose tissue through the mineralocorticoid receptor pathway. *FASEB Journal*.

[69] Gaida M., Dapunt U., Hansch G. M. (2016). Sensing developing biofilms: the bitter receptor T2R38 on myeloid cells. *Pathogens and Disease*.

[70] Hughes S., Magnay J., Foreman M., Publicover S. J., Dobson J. P., El Haj A. J. (2006). Expression of the mechanosensitive 2PK+ channel TREK-1 in human osteoblasts. *Journal of Cellular Physiology*.

[71] Li X., Dong X., Zheng S., Xiao J. (2013). Expression and localization of TASK-1, -2 and -3 channels in MG63 human osteoblast-like cells. *Oncology Letters*.

[72] Mobasheri A., Barrett-Jolley R., Shakibaei M., Canessa C. M., Martin-Vasallo P., Kamkin A., Kiseleva I. (2005). *Enigmatic Roles of the Epithelial Sodium Channel (ENaC) in Articular Chondrocytes and Osteoblasts: Mechanotransduction, Sodium Transport or Extracellular Sodium Sensing?*.

[73] Lu L., Wu L., Jia H. (2012). The epithelial sodium channel is involved in dexamethasone-induced osteoblast differentiation and mineralization. *Cell Biology and Toxicology*.

[74] Smith M., Wilson R., O'Brien S., Tufarelli C., Anderson S. I., O'Sullivan S. E. (2015). The effects of the endocannabinoids anandamide and 2-arachidonoylglycerol on human osteoblast proliferation and differentiation. *PLoS ONE*.

[75] Lund T. C., Kobs A. J., Kramer A. (2013). Bone marrow stromal and vascular smooth muscle cells have chemosensory capacity via bitter taste receptor expression. *PLoS ONE*.

[76] Kanczler J. M., Sura H. S., Magnay J. (2010). Controlled differentiation of human bone marrow stromal cells using magnetic nanoparticle technology. *Tissue Engineering—Part A*.

[77] Zou W., Lin H., Liu W. (2016). Moxibustion relieves visceral hyperalgesia via inhibition of transient receptor potential vanilloid 1 (TRPV1) and heat shock protein (HSP) 70 expression in rat bone marrow cells. *Acupuncture in Medicine*.

[78] Trujillo E., Alvarez de la Rosa D., Mobasheri et al. A. (1999). Sodium transport systems in human chondrocytes. II. Expression of ENaC, Na+/K+/2Cl- cotransporter and Na+/H+ exchangers in healthy and arthritic chondrocytes. *Histology and Histopathology*.

[79] O'Conor C. J., Leddy H. A., Benefield H. C., Liedtke W. B., Guilak F. (2014). TRPV4-mediated mechanotransduction regulates the metabolic response of chondrocytes to dynamic loading. *Proceedings of the National Academy of Sciences of the United States of America*.

[80] Malki A., Fiedler J., Fricke K., Ballweg I., Pfaffl M. W., Krautwurst D. (2015). Class I odorant receptors, TAS1R and TAS2R taste receptors, are markers for subpopulations of circulating leukocytes. *Journal of Leukocyte Biology*.

[81] Max M., Shanker Y. G., Huang L. (2001). Tas1r3, encoding a new candidate taste receptor, is allelic to the sweet responsiveness locus Sac. *Nature Genetics*.

[82] Maurer S., Wabnitz G. H., Kahle N. A. (2015). Tasting *Pseudomonas aeruginosa* biofilms: human neutrophils express the bitter receptor T2R38 as sensor for the quorum sensing molecule N-(3-oxododecanoyl)-l-homoserine lactone. *Frontiers in Immunology*.

[83] Nam J. H., Shin D. H., Zheng H. (2011). Expression of TASK-2 and its upregulation by B cell receptor stimulation in WEHI-231 mouse immature B cells. *American Journal of Physiology—Cell Physiology*.

[84] Yin J., Michalick L., Tang C. (2016). Role of transient receptor potential vanilloid 4 in neutrophil activation and acute lung injury. *American Journal of Respiratory Cell and Molecular Biology*.

[85] Mosinger B., Redding K. M., Parker M. R. (2013). Genetic loss or pharmacological blockade of testes-expressed taste genes causes male sterility. *Proceedings of the National Academy of Sciences of the United States of America*.

[86] Li F., Zhou M. (2012). Depletion of bitter taste transduction leads to massive spermatid loss in transgenic mice. *Molecular Human Reproduction*.

[87] Chow G. E., Muller C. H., Curnow E. C., Hayes E. S. (2007). Expression of two-pore domain potassium channels in nonhuman primate sperm. *Fertility and Sterility*.

[88] Kumar P. G., Shoeb M. (2011). The role of TRP ion channels in testicular function. *Advances in Experimental Medicine and Biology*.

[89] Richter M., Tscheudschilsuren G., Eschke D., Aust G., Spanel-Borowski K., Nieber K. (2002). Voltage-dependent potassium channels in cytokeratin-positive and cytokeratin-negative microvascular endothelial cells of the corpus luteum. *Cell and Tissue Research*.

[90] Lizanecz E., Bagi Z., Pasztor E. T. (2006). Phosphorylation-dependent desensitization by anandamide of vanilloid receptor-1 (TRPV1) function in rat skeletal muscle arterioles and in Chinese hamster ovary cells expressing TRPV1. *Molecular Pharmacology*.

[91] Teilmann S. C., Byskov A. G., Pedersen P. A., Wheatley D. N., Pazour G. J., Christensen S. T. (2005). Localization of transient receptor potential ion channels in primary and motile cilia of the female murine reproductive organs. *Molecular Reproduction and Development*.

[92] Ruan Y. C., Guo J. H., Liu X. (2012). Activation of the epithelial Na+ channel triggers prostaglandin E2 release and production required for embryo implantation. *Nature Medicine*.

[93] Pohoczky K., Kun J., Szalontai B. (2016). Estrogen-dependent up-regulation of TRPA1 and TRPV1 receptor proteins in the rat endometrium. *Journal of Molecular Endocrinology*.

[94] Singh N., Chakraborty R., Bhullar R. P., Chelikani P. (2014). Differential expression of bitter taste receptors in non-cancerous breast epithelial and breast cancer cells. *Biochemical and Biophysical Research Communications*.

[95] Kamikawa A., Ishikawa T. (2014). Functional expression of a Kir2.1-like inwardly rectifying potassium channel in mouse mammary secretory cells. *American Journal of Physiology—Cell Physiology*.

[96] So Y. L., Palmer M. L., Maniak P. J., Soo H. J., Pan D. R., O'Grady S. M. (2007). P2Y receptor regulation of sodium transport in human mammary epithelial cells. *American Journal of Physiology—Cell Physiology*.

[97] Wu T. T. L., Peters A. A., Tan P. T., Roberts-Thomson S. J., Monteith G. R. (2014). Consequences of activating the calcium-permeable ion channel TRPV1 in breast cancer cells with regulated TRPV1 expression. *Cell Calcium*.

[98] Wolfle U., Elsholz F. A., Kersten A., Haarhaus B., Müller W. E., Schempp C. M. (2015). Expression and functional activity of the bitter taste receptors TAS2R1 and TAS2R38 in human keratinocytes. *Skin Pharmacology and Physiology*.

[99] Kim D., Fujita A., Horio Y., Kurachi Y. (1998). Cloning and functional expression of a novel cardiac two-pore background K+ channel (cTBAK-1). *Circulation Research*.

[100] Brouard M., Casado M., Djelidi S., Barrandon Y., Farman N. (1999). Epithelial sodium channel in human epidermal keratinocytes: expression of its subunits and relation to sodium transport and differentiation. *Journal of Cell Science*.

[101] Yang Y. S., Cho S. I., Choi M. G. (2015). Increased expression of three types of transient receptor potential channels (TRPA1, TRPV4 and TRPV3) in burn scars with postburn pruritus. *Acta Dermato-Venereologica*.

[102] Kida N., Sokabe T., Kashio M. (2012). Importance of transient receptor potential vanilloid 4 (TRPV4) in epidermal barrier function in human skin keratinocytes. *Pflugers Archiv European Journal of Physiology*.

[103] Clark A. A., Dotson C. D., Elson A. E. T. (2015). TAS2R bitter taste receptors regulate thyroid function. *FASEB Journal*.

[104] Kim D., Gnatenco C. (2001). TASK-5, a new member of the tandem-pore K+ channel family. *Biochemical and Biophysical Research Communications*.

[105] Verrier B., Champigny G., Barbry P., Gerard C., Mauchamp J., Lazdunski M. (1989). Identification and properties of a novel type of Na+‐permeable amiloride‐sensitive channel in thyroid cells. *European Journal of Biochemistry*.

[106] Salinas M., Reyes R., Lesage F. (1999). Cloning of a new mouse two-P domain channel subunit and a human homologue with a unique pore structure. *Journal of Biological Chemistry*.

[107] Grimm E. R., Steinle N. I. (2011). Genetics of eating behavior: established and emerging concepts. *Nutrition Reviews*.

[108] Czirjak G. (2000). TASK (TWIK-related acid-sensitive K+ channel) is expressed in glomerulosa cells of rat adrenal cortex and inhibited by Angiotensin II. *Molecular Endocrinology*.

[109] Sharma R., Dash B. (2006). *Caraka Samhita*.

[110] Stefan E. (2005). *Astanga Hrdayam/Vagbhata*.

[111] Zostawa J., Adamczyk J., Sowa P., Adamczyk-Sowa M. (2017). The influence of sodium on pathophysiology of multiple sclerosis. *Neurological Sciences*.

[112] Luo T., Ji W.-J., Yuan F. (2016). Th17/Treg imbalance induced by dietary salt variation indicates inflammation of target organs in humans. *Scientific Reports*.

[113] Wei Y., Lu C., Chen J. (2016). High salt diet stimulates gut Th17 response and exacerbates TNBS-induced colitis in mice. *Oncotarget*.

[114] Alharbi B. M., Tso M. K., Macdonald R. L. (2016). Animal models of spontaneous intracerebral hemorrhage. *Neurological Research*.

[115] MacGregor G. (1986). Salt and hypertension.. *British Journal of Clinical Pharmacology*.

[116] Loh J. T., Gaddy J. A., Scott Algood H. M., Gaudieri S., Mallal S., Cover T. L. (2015). Helicobacter pylori adaptation in vivo in response to a high-salt diet. *Infection and Immunity*.

[117] Wiig H., Schröder A., Neuhofer W. (2013). Immune cells control skin lymphatic electrolyte homeostasis and blood pressure. *Journal of Clinical Investigation*.

[118] Fellner R. C., Cook A. K., O'Connor P. M., Zhang S., Pollock D. M., Inscho E. W. (2014). High-salt diet blunts renal autoregulation by a reactive oxygen species-dependent mechanism. *American Journal of Physiology—Renal Physiology*.

[119] Pandey G. (2005). *Dravyaguna vijnana*.

[120] Dallmeier K., Carlini E. A. (1981). Anesthetic, hypothermic, myorelaxant and anticonvulsant effects of synthetic eugenol derivatives and natural analogues. *Pharmacology*.

[121] Talavera K., Yasumatsu K., Voets T. (2005). Heat activation of TRPM5 underlies thermal sensitivity of sweet taste. *Nature*.

[122] Mancuso G., Borgonovo G., Scaglioni L., Bassoli A. (2015). Phytochemicals from *Ruta graveolens* activate TAS2R bitter taste receptors and TRP channels involved in gustation and nociception. *Molecules*.

[123] Peier A. M., Moqrich A., Hergarden A. C. (2002). A TRP channel that senses cold stimuli and menthol. *Cell*.

[124] Latorre R., Brauchi S., Orta G., Zaelzer C., Vargas G. (2007). ThermoTRP channels as modular proteins with allosteric gating. *Cell Calcium*.

[125] Vetter I., Lewis R. J. (2011). Natural product ligands of TRP channels. *Advances in Experimental Medicine and Biology*.

[126] Macpherson L. J., Hwang S. W., Miyamoto T., Dubin A. E., Patapoutian A., Story G. M. (2006). More than cool: *Promiscuous* relationships of menthol and other sensory compounds. *Molecular and Cellular Neuroscience*.

[127] Story G. M., Peier A. M., Reeve A. J. (2003). ANKTM1, a TRP-like channel expressed in nociceptive neurons, is activated by cold temperatures. *Cell*.

[128] Bandell M., Story G. M., Hwang S. W. (2004). Noxious cold ion channel TRPA1 is activated by pungent compounds and bradykinin. *Neuron*.

[129] Güler A. D., Lee H., Iida T., Shimizu I., Tominaga M., Caterina M. (2002). Heat-evoked activation of the ion channel, TRPV4. *Journal of Neuroscience*.

[130] Smith P. L., Maloney K. N., Pothen R. G., Clardy J., Clapham D. E. (2006). Bisandrographolide from *Andrographis paniculata* activates TRPV4 channels. *The Journal of Biological Chemistry*.

[131] Peixoto-Neves D., Wang Q., Leal-Cardoso J. H., Rossoni L. V., Jaggar J. H. (2015). Eugenol dilates mesenteric arteries and reduces systemic BP by activating endothelial cell TRPV4 channels. *British Journal of Pharmacology*.

[132] Marwaha L., Bansal Y., Singh R., Saroj P., Bhandari R., Kuhad A. (2016). TRP channels: potential drug target for neuropathic pain. *Inflammopharmacology*.

[133] Xu H., Delling M., Jun J. C., Clapham D. E. (2006). Oregano, thyme and clove-derived flavors and skin sensitizers activate specific TRP channels. *Nature Neuroscience*.

[134] Moqrich A., Hwang S. W., Earley T. J. (2005). Impaired thermosensation in mice lacking TRPV3, a heat and camphor sensor in the skin. *Science*.

[135] Sokabe T., Tominaga M. (2009). Molecular mechanisms underlying thermosensation in mammals. *Brain and Nerve*.

[136] Caterina M. J., Schumacher M. A., Tominaga M., Rosen T. A., Levine J. D., Julius D. (1997). The capsaicin receptor: a heat-activated ion channel in the pain pathway. *Nature*.

[137] McNamara F. N., Randall A., Gunthorpe M. J. (2005). Effects of piperine, the pungent component of black pepper, at the human vanilloid receptor (TRPV1). *British Journal of Pharmacology*.

[138] Tominaga M., Caterina M. J., Malmberg A. B. (1998). The cloned capsaicin receptor integrates multiple pain-producing stimuli. *Neuron*.

[139] De Petrocellis L., Ligresti A., Moriello A. S. (2011). Effects of cannabinoids and cannabinoid-enriched *Cannabis* extracts on TRP channels and endocannabinoid metabolic enzymes. *British Journal of Pharmacology*.

[140] Qin N., Neeper M. P., Liu Y., Hutchinson T. L., Lubin M. L., Flores C. M. (2008). TRPV2 is activated by cannabidiol and mediates CGRP release in cultured rat dorsal root *Ganglion neurons*. *Journal of Neuroscience*.

[141] Macpherson L. J., Geierstanger B. H., Viswanath V. (2005). The pungency of garlic: activation of TRPA1 and TRPV1 in response to allicin. *Current Biology*.

[142] Kim Y.-S., Hong C. S., Lee S. W., Nam J. H., Kim B. J. (2016). Effects of ginger and its pungent constituents on transient receptor potential channels. *International Journal of Molecular Medicine*.

[143] Jordt S.-E., Bautista D. M., Chuang H.-H. (2004). *Mustard oils* and *cannabinoids* excite sensory nerve fibres through the TRP channel ANKTM1. *Nature*.

[144] Tsuchiya K., Kubota K., Ohbuchi K. (2016). Transient receptor potential ankyrin 1 agonists improve intestinal transit in a murine model of postoperative ileus. *Neurogastroenterology and Motility*.

[145] Peyrot des Gachons C., Uchida K., Bryant B. (2011). Unusual pungency from extra-virgin olive oil is attributable to restricted spatial expression of the receptor of oleocanthal. *Journal of Neuroscience*.

[146] Wang Y. Y., Chang R. B., Liman E. R. (2010). TRPA1 is a component of the nociceptive response to CO_2_. *Journal of Neuroscience*.

[147] Escalera J., Von Hehn C. A., Bessac B. F., Sivula M., Jordt S.-E. (2008). TRPA1 mediates the noxious effects of natural sesquiterpene deterrents. *Journal of Biological Chemistry*.

[148] Bassoli A., Borgonovo G., Caimi S. (2009). Taste-guided identification of high potency TRPA1 agonists from *Perilla frutescens*. *Bioorganic and Medicinal Chemistry*.

[149] Nelson G., Hoon M. A., Chandrashekar J., Zhang Y., Ryba N. J. P., Zuker C. S. (2001). Mammalian sweet taste receptors. *Cell*.

[150] Laffitte A., Neiers F., Briand L. (2014). Functional roles of the sweet taste receptor in oral and extraoral tissues. *Current Opinion in Clinical Nutrition and Metabolic Care*.

[151] Behrens M., Meyerhof W. (2011). Gustatory and extragustatory functions of mammalian taste receptors. *Physiology and Behavior*.

[152] Behrens M., Meyerhof W. (2006). Bitter taste receptors and human bitter taste perception. *Cellular and Molecular Life Sciences*.

[153] Wiener A., Shudler M., Levit A., Niv M. Y. (2012). BitterDB: a database of bitter compounds. *Nucleic Acids Research*.

[154] Rodgers S., Busch J., Peters H., Christ-Hazelhof E. (2005). Building a tree of knowledge: analysis of bitter molecules. *Chemical Senses*.

[155] Meyerhof W., Batram C., Kuhn C. (2009). The molecular receptive ranges of human TAS2R bitter taste receptors. *Chemical Senses*.

[156] Behrens M., Korsching S. I., Meyerhof W. (2014). Tuning properties of avian and frog bitter taste receptors dynamically fit gene repertoire sizes. *Molecular Biology and Evolution*.

[157] Lossow K., Hubner S., Roudnitzky N. (2016). Comprehensive analysis of mouse bitter taste receptors reveals different molecular receptive ranges for orthologous receptors in mice and humans. *Journal of Biological Chemistry*.

[158] Zhang Y., Hoon M. A., Chandrashekar J. (2003). Coding of sweet, bitter, and umami tastes: different receptor cells sharing similar signaling pathways. *Cell*.

[159] Gilca M., Barbulescu A. (2015). Taste of medicinal plants: a potential tool in predicting ethnopharmacological activities?. *Journal of Ethnopharmacology*.

[160] Behrens M., Meyerhof W. (2015). *Flavour Development, Analysis and Perception in Food and Beverages*.

[161] Saites L. N., Goldsmith Z., Densky J., Guedes V. A., Boughter J. D. J. (2015). Mice perceive synergistic umami mixtures as tasting sweet. *Chemical Senses*.

[162] Kusuhara Y., Yoshida R., Ohkuri T. (2013). Taste responses in mice lacking taste receptor subunit T1R1. *The Journal of Physiology*.

[163] Susruta and K. K. L. Bhishagratna (translator) (1998). *Susruta Samhita*.

[164] Iwata S., Yoshida R., Ninomiya Y. (2014). Taste transductions in taste receptor cells: basic tastes and moreover. *Current Pharmaceutical Design*.

[165] Galindo M. M., Voigt N., Stein J. (2012). G protein-coupled receptors in human fat taste perception. *Chemical Senses*.

[166] Cartoni C., Yasumatsu K., Ohkuri T. (2010). Taste preference for fatty acids is mediated by GPR40 and GPR120. *The Journal of Neuroscience*.

[167] Febbraio M., Hajjar D. P., Silverstein R. L. (2001). CD36: a class B scavenger receptor involved in angiogenesis, atherosclerosis, inflammation, and lipid metabolism. *Journal of Clinical Investigation*.

[168] Besnard P., Passilly-Degrace P., Khan N. A. (2015). Taste of fat: a sixth taste modality?. *Physiological Reviews*.

[169] Ozdener M. H., Subramaniam S., Sundaresan S. (2014). CD36- and GPR120-mediated Ca^2+^ signaling in human taste bud cells mediates differential responses to fatty acids and is altered in obese mice. *Gastroenterology*.

[170] Martin C., Passilly-Degrace P., Gaillard D., Merlin J.-F., Chevrot M., Besnard P. (2011). The lipid-sensor candidates CD36 and GPR120 are differentially regulated by dietary lipids in mouse taste buds: impact on spontaneous fat preference. *PLoS ONE*.

[171] Ren Z. J., Rhyu M.-R., Phan T.-H. T. (2013). TRPM5-dependent amiloride- and benzamil-insensitive NaCl chorda tympani taste nerve response. *American Journal of Physiology—Gastrointestinal and Liver Physiology*.

[172] Sclafani A., Ackroff K. (2012). Role of gut nutrient sensing in stimulating appetite and conditioning food preferences. *American Journal of Physiology—Regulatory Integrative and Comparative Physiology*.

[173] Lim J., Lawless H. T. (2005). Oral sensations from iron and copper sulfate. *Physiology and Behavior*.

[174] des Gachons C. P., Mura E., Speziale C., Favreau C. J., Dubreuil G. F., Breslin P. A. S. (2012). Opponency of astringent and fat sensations. *Current Biology*.

[175] Jiang Y., Gong N. N., Matsunami H. (2014). Astringency: A more stringent definition. *Chemical Senses*.

[176] Schiffman S. S., Suggs M. S., Sostman L., Simon S. A. (1992). Chorda tympani and lingual nerve responses to astringent compounds in rodents. *Physiology and Behavior*.

[177] Schobel N., Radtke D., Lubbert M. (2012). Trigeminal ganglion neurons of mice show intracellular chloride accumulation and chloride-dependent amplification of capsaicin-induced responses. *PLoS ONE*.

[178] Luck G., Liao H., Murray N. J. (1994). Polyphenols, astringency and proline-rich proteins. *Phytochemistry*.

[179] Roper S. D. (2015). The taste of table salt. *Pflugers Archiv*.

[180] Vandenbeuch A., Clapp T. R., Kinnamon S. C. (2008). Amiloride-sensitive channels in type I fungiform taste cells in mouse. *BMC Neuroscience*.

[181] Oka Y., Butnaru M., Von Buchholtz L., Ryba N. J. P., Zuker C. S. (2013). High salt recruits aversive taste pathways. *Nature*.

[182] Heck G. L., Mierson S., Desimone J. A. (1984). Salt taste transduction occurs through an amiloride-sensitive sodium transport pathway. *Science*.

[183] Stähler F., Riedel K., Demgensky S. (2008). A role of the epithelial sodium channel in human salt taste transduction?. *Chemosensory Perception*.

[184] DeSimone J. A., Lyall V. (2006). Taste receptors in the gastrointestinal tract III. Salty and sour taste: sensing of sodium and protons by the tongue. *American Journal of Physiology—Gastrointestinal and Liver Physiology*.

[185] Ye W., Chang R. B., Bushman et al. J. D. (2016). The K+ channel KIR2.1 functions in tandem with proton influx to mediate sour taste transduction. *Proceedings of the National Academy of Sciences of the United States of America*.

[186] Roper S. D. (2007). Signal transduction and information processing in mammalian taste buds. *Pflugers Archiv*.

[187] Ishimaru Y., Inada H., Kubota M., Zhuang H., Tominaga M., Matsunami H. (2006). Transient receptor potential family members PKD1L3 and PKD2L1 form a candidate sour taste receptor. *Proceedings of the National Academy of Sciences of the United States of America*.

[188] Kataoka S., Yang R., Ishimaru Y. (2008). The candidate sour taste receptor, PKD2L1, Is expressed by type III taste cells in the mouse. *Chemical Senses*.

[189] Richter T. A., Dvoryanchikov G. A., Chaudhari N., Roper S. D. (2004). Acid-sensitive two-pore domain potassium (K2P) channels in mouse taste buds. *Journal of Neurophysiology*.

[190] Lin W., Burks C. A., Hansen D. R., Kinnamon S. C., Gilbertson T. A. (2004). Taste receptor cells express pH-sensitive leak K+ channels. *Journal of Neurophysiology*.

[191] Challis R. C., Ma M. (2016). Sour taste finds closure in a potassium channel. *Proceedings of the National Academy of Sciences of the United States of America*.

[192] Talley E. M., Sirois J. E., Lei Q., Bayliss D. A. (2003). Two-pore-domain (KCNK) potassium channels: dynamic roles in neuronal function. *Neuroscientist*.

[193] O'Connell A. D., Morton M. J., Hunter M. (2002). Two-pore domain K+ channels—Molecular sensors. *Biochimica et Biophysica Acta—Biomembranes*.

[194] Chavez R. A., Gray A. T., Zhao B. B. (1999). TWIK-2, a new weak inward rectifying member of the tandem pore domain potassium channel family. *Journal of Biological Chemistry*.

[195] Kurogi M., Miyashita M., Emoto Y., Kubo Y., Saitoh O. (2012). Green tea polyphenol epigallocatechin gallate activates trpa1 in an intestinal enteroendocrine cell Line, STC-1. *Chemical Senses*.

[196] Sbarbati A., Osculati F. (2005). The taste cell-related diffuse chemosensory system. *Progress in Neurobiology*.

[197] Sbarbati A., Bramanti P., Benati D., Merigo F. (2010). The diffuse chemosensory system: exploring the iceberg toward the definition of functional roles. *Progress in Neurobiology*.

[198] Osculati F., Bentivoglio M., Castellucci M., Cinti S., Zancanaro C., Sbarbati A. (2007). The solitary chemosensory cells and the diffuse chemosensory system of the airway. *European Journal of Histochemistry*.

[199] Sharma P., Panebra A., Pera T. (2016). Antimitogenic effect of bitter taste receptor agonists on airway smooth muscle cells. *American Journal of Physiology—Lung Cellular and Molecular Physiology*.

[200] Grassin-Delyle S., Naline E., Devillier P. (2015). Taste receptors in asthma. *Current Opinion in Allergy and Clinical Immunology*.

[201] Lee R. J., Cohen N. A. (2015). Taste receptors in innate immunity. *Cellular and Molecular Life Sciences*.

[202] Smith D. V., Lemon C. H. (2007). *Neural Coding in the rNST*.

[203] Rath S., Panja A., Shinde A., Nagar L. (2014). The scientific basis of rasa (taste) of a substance as a tool to explore its pharmacological behavior. *Ancient Science of Life*.

[204] Pushpan R., Nishtewsar K. (2014). Rasa Nirdhārana (assessment of taste) of *Leonotis nepetifolia* (L.) R. Br.: a preliminary study in healthy volunteers. *Ancient Science of Life*.

[205] Leslie C., Young A., D. C. American Anthropological Association (1992). *Paths to Asian Medical Knowledge*.

[206] Nam J. K. U. (2014). Medieval European medicine and Asian spices. *Uisahak*.

[207] Messer E. (1987). The hot and cold in mesoamerican indigenous and hispanicized thought. *Social Science and Medicine*.

[208] Geck M. S., Cabras S., Casu L., Reyes Garcia A. J., Leonti M. (2017). The taste of heat: how humoral qualities act as a cultural filter for chemosensory properties guiding herbal medicine. *Journal of Ethnopharmacology*.

[209] de Medeiros P. M., Santos Pinto B. L., do Nascimento V. T. (2015). Can organoleptic properties explain the differential use of medicinal plants? Evidence from Northeastern Brazil. *Journal of Ethnopharmacology*.

[210] Leonti M., Sticher O., Heinrich M. (2002). Medicinal plants of the Popoluca, México: organoleptic properties as indigenous selection criteria. *Journal of Ethnopharmacology*.

[211] Qadri Y. J., Rooj A. K., Fuller C. M. (2012). ENaCs and ASICs as therapeutic targets. *American Journal of Physiology—Cell Physiology*.

[212] Jang H.-J., Kokrashvili Z., Theodorakis M. J. (2007). Gut-expressed gustducin and taste receptors regulate secretion of glucagon-like peptide-1. *Proceedings of the National Academy of Sciences of the United States of America*.

[213] Dazert E., Hall M. N. (2011). mTOR signaling in disease. *Current Opinion in Cell Biology*.

[214] Bortvedt S. F., Lund P. K. (2012). Insulin-like growth factor 1: common mediator of multiple enterotrophic hormones and growth factors. *Current Opinion in Gastroenterology*.

[215] Aburto M. R., Magariños M., Leon Y., Varela-Nieto I., Sanchez-Calderon H. (2012). AKT signaling mediates IGF-I survival actions on otic neural progenitors. *PLoS ONE*.

[216] Murovets V. O., Bachmanov A. A., Zolotarev V. A. (2015). Impaired glucose metabolism in mice lacking the Tas1r3 taste receptor gene. *PLoS ONE*.

[217] Shin Y.-J., Park J.-H., Choi J.-S., Chun M.-H., Moon Y. W., Lee M.-Y. (2010). Enhanced expression of the sweet taste receptors and alpha-gustducin in reactive astrocytes of the rat hippocampus following ischemic injury. *Neurochemical Research*.

[218] Simon B. R., Learman B. S., Parlee S. D. (2014). Sweet taste receptor deficient mice have decreased adiposity and increased bone mass. *PLoS ONE*.

[219] Kokabu S., Lowery J. W., Toyono T. (2015). Muscle regulatory factors regulate T1R3 taste receptor expression. *Biochemical and Biophysical Research Communications*.

[220] Wauson E. M., Zaganjor E., Lee A.-Y. (2012). The G protein-coupled taste receptor T1R1/T1R3 regulates mTORC1 and autophagy. *Molecular Cell*.

[221] Gong T., Wei Q., Mao D., Shi F. (2016). Expression patterns of taste receptor type 1 subunit 3 and *α*-gustducin in the mouse testis during development. *Acta Histochemica*.

[222] Meyer D., Voigt A., Widmayer P. (2012). Expression of tas1 taste receptors in mammalian spermatozoa: functional role of Tas1r1 in regulating basal Ca^2+^ and camp concentrations in spermatozoa. *PLoS ONE*.

[223] Maillet E. L., Margolskee R. F., Mosinger B. (2009). Phenoxy herbicides and fibrates potently inhibit the human chemosensory receptor subunit T1R3. *Journal of Medicinal Chemistry*.

[224] Hellekant G., Schmolling J., Marambaud P., Rose-Hellekant T. A. (2015). CALHM1 deletion in mice affects glossopharyngeal taste responses, food intake, body weight, and life span. *Chemical Senses*.

[225] Morris J. Z., Tissenbaum H. A., Ruvkun G. (1996). A phosphatidylinositol-3-OH kinase family member regulating longevity and diapause in *Caenorhabditis elegans*. *Nature*.

[226] Kimura K. D., Tissenbaum H. A., Liu Y., Ruvkun G. (1997). Daf-2, an insulin receptor-like gene that regulates longevity and diapause in *Caenorhabditis elegans*. *Science*.

[227] Ostojic I., Boll W., Waterson M. J. (2014). Positive and negative gustatory inputs affect *Drosophila lifespan* partly in parallel to dFOXO signaling. *Proceedings of the National Academy of Sciences of the United States of America*.

[228] Shin Y.-K., Cong W.-N., Cai H. (2012). Age-related changes in mouse taste bud morphology, hormone expression, and taste responsivity. *Journals of Gerontology - Series A Biological Sciences and Medical Sciences*.

[229] Cronin H., Draelos Z. D. (2010). Top 10 botanical ingredients in 2010 anti-aging creams. *Journal of Cosmetic Dermatology*.

[230] Simon B. R., Parlee S. D., Learman B. S. (2013). Artificial sweeteners stimulate adipogenesis and suppress lipolysis independently of sweet taste receptors. *Journal of Biological Chemistry*.

[231] Matsumoto Y., Matsuura T., Aoyagi H. (2013). Antiviral activity of glycyrrhizin against hepatitis C virus in vitro. *PLoS ONE*.

[232] Avau B., Bauters D., Steensels S. (2015). The gustatory signaling pathway and bitter taste receptors affect the development of obesity and adipocyte metabolism in mice. *PLoS ONE*.

[233] Gupta N., Tiwari R. (2015). An Ayurvedic approach to hypercholesterolemia. *International Ayurvedic Medical Journal*.

[234] Drewnowski A., Gomez-Carneros C. (2000). Bitter taste, phytonutrients, and the consumer: a review. *American Journal of Clinical Nutrition*.

[235] Sparks J. T., Dickens J. C. (2016). Bitter-sensitive gustatory receptor neuron responds to chemically diverse insect repellents in the common malaria mosquito *Anopheles quadrimaculatus*. *Naturwissenschaften*.

[236] Janssen S., Laermans J., Verhulst P.-J., Thijs T., Tack J., Depoortere I. (2011). Bitter taste receptors and *α*-gustducin regulate the secretion of ghrelin with functional effects on food intake and gastric emptying. *Proceedings of the National Academy of Sciences of the United States of America*.

[237] Avau B., Rotondo A., Thijs T. (2015). Targeting extra-oral bitter taste receptors modulates gastrointestinal motility with effects on satiation. *Scientific Reports*.

[238] Glendinning J. I. (1994). Is the bitter rejection response always adaptive?. *Physiology and Behavior*.

[239] Drewnowski A., Henderson S. A., Shore A. B. (1997). Taste responses to naringin, a flavonoid, and the acceptance of grapefruit juice are related to genetic sensitivity to 6-n-propylthiouracil. *American Journal of Clinical Nutrition*.

[240] Drewnowski A. (2002). Genetic markers, taste responses, and food preferences. *Chemistry of Taste*.

[241] Olson J. M., Boehnke M., Neiswanger K., Roche A. F., Siervogel R. M., MacCluer J. W. (1989). Alternative genetic models for the inheritance of the phenylthiocarbamide taste deficiency. *Genetic Epidemiology*.

[242] Fahey J. W., Zhang Y., Talalay P. (1997). *Broccoli sprouts*: an exceptionally rich source of inducers of enzymes that protect against chemical carcinogens. *Proceedings of the National Academy of Sciences of the United States of America*.

[243] Warburg O. (1956). On the origin of cancer cells. *Science*.

[244] Luc R., Tortorella S. M., Ververis K., Karagiannis T. C. (2015). Lactate as an insidious metabolite due to the Warburg effect. *Molecular Biology Reports*.

[245] Ngo H., Tortorella S. M., Ververis K., Karagiannis T. C. (2015). The Warburg effect: molecular aspects and therapeutic possibilities. *Molecular biology reports*.

[246] Sharma S. H., Thulasingam S., Chellappan D. R., Chinnaswamy P., Nagarajan S. (2017). Morin and Esculetin supplementation modulates c-myc induced energy metabolism and attenuates neoplastic changes in rats challenged with the procarcinogen 1,2-dimethylhydrazine. *European Journal of Pharmacology*.

[247] Chen V., Staub R. E., Baggett S. (2012). Identification and analysis of the active phytochemicals from the anti-cancer botanical extract Bezielle. *PLoS ONE*.

[248] Harmon A. W., Patel Y. M. (2004). Naringenin inhibits glucose uptake in MCF-7 breast cancer cells: a mechanism for impaired cellular proliferation. *Breast Cancer Research and Treatment*.

[249] Martel F., Guedes M., Keating E. (2016). Effect of polyphenols on glucose and lactate transport by breast cancer cells. *Breast Cancer Research and Treatment*.

[250] Moreira L., Araujo I., Costa T. (2013). Quercetin and epigallocatechin gallate inhibit glucose uptake and metabolism by breast cancer cells by an estrogen receptor-independent mechanism. *Experimental Cell Research*.

[251] Yang Y., Wolfram J., Boom K., Fang X., Shen H., Ferrari M. (2013). Hesperetin impairs glucose uptake and inhibits proliferation of breast cancer cells. *Cell Biochemistry and Function*.

[252] Neri M., Fineschi V., Di Paolo M. (2015). Cardiac oxidative stress and inflammatory cytokines response after myocardial infarction. *Current Vascular Pharmacology*.

[253] Faria A., Persaud S. J. (2016). Cardiac oxidative stress in diabetes: mechanisms and therapeutic potential. *Pharmacology & Therapeutics*.

[254] Piasecka A., Jedrzejczak-Rey N., Bednarek P. (2015). Secondary metabolites in plant innate immunity: conserved function of divergent chemicals. *New Phytologist*.

[255] Harborne J. B. (2001). Twenty-five years of chemical ecology. *Natural Product Reports*.

[256] Lee R. J., Kofonow J. M., Rosen P. L. (2014). Bitter and sweet taste receptors regulate human upper respiratory innate immunity. *The Journal of Clinical Investigation*.

[257] Lee R. J., Cohen N. A. (2013). The emerging role of the bitter taste receptor T2R38 in upper respiratory infection and chronic rhinosinusitis. *American Journal of Rhinology and Allergy*.

[258] Gil S., Coldwell S., Drury J. L. (2015). Genotype-specific regulation of oral innate immunity by T2R38 taste receptor. *Molecular Immunology*.

[259] Lee R. J., Cohen N. A. (2015). Role of the bitter taste receptor T2R38 in upper respiratory infection and chronic rhinosinusitis. *Current Opinion in Allergy and Clinical Immunology*.

[260] Deshpande D. A., Wang W. C. H., McIlmoyle et al. E. L. (2010). Bitter taste receptors on airway smooth muscle bronchodilate by localized calcium signaling and reverse obstruction. *Journal of Natural Medicines*.

[261] Leonard W. R. (2002). Food for thought. Dietary change was a driving force in human evolution. *Scientific American*.

[262] Brockhoff A., Behrens M., Roudnitzky N., Appendino G., Avonto C., Meyerhof W. (2011). Receptor agonism and antagonism of dietary bitter compounds. *Journal of Neuroscience*.

[263] Belitz H. D., Wieser H. (1985). Bitter compounds: occurrence and structure-Activity relationships. *Food Reviews International*.

[264] Tierson F. D., Olsen C. L., Hook E. B. (1986). Nausea and vomiting of pregnancy and association with pregnancy outcome. *American Journal of Obstetrics and Gynecology*.

[265] Little R. E. (1980). Maternal alcohol and tobacco use and nausea and vomiting during pregnancy: relation to infant birthweight. *Acta Obstetricia et Gynecologica Scandinavica*.

[266] Weigel R. M., Weigel M. M. (1989). Nausea and vomiting of early pregnancy and pregnancy outcome. A meta‐analytical review. *British Journal of Obstetrics and Gynaecology*.

[267] Weigel M. M., Weigel R. M. (1989). Nausea and vomiting of early pregnancy and pregnancy outcome. An epidemiological study. *British Journal of Obstetrics and Gynaecology*.

[268] Breslin P. A. S. (2013). An evolutionary perspective on food and human taste. *Current Biology*.

[269] Lu P., Zhang C., Lifshitz L. M., ZhuGe R. (2017). Extraoral bitter taste receptors in health and disease. *The Journal of General Physiology*.

[270] Mennella J. A., Spector A. C., Reed D. R., Coldwell S. E. (2013). The bad taste of medicines: overview of basic research on bitter taste. *Clinical Therapeutics*.

[271] Pavlov T. S., Levchenko V., Ilatovskaya D. V., Palygin O., Staruschenko A. (2015). Impaired epithelial Na^+^ channel activity contributes to cystogenesis and development of autosomal recessive polycystic kidney disease in PCK rats. *Pediatric Research*.

[272] Lee S. H., Somlo S. (2014). Cyst growth, polycystins, and primary cilia in autosomal dominant polycystic kidney disease. *Kidney Research and Clinical Practice*.

[273] Bondarava M., Li T., Endl E., Wehner F. (2009). *α*-ENaC is a functional element of the hypertonicity-induced cation channel in HepG2 cells and it mediates proliferation. *Pflugers Archiv*.

[274] Zheleznova N. N., Wilson P. D., Staruschenko A. (2011). Epidermal growth factor-mediated proliferation and sodium transport in normal and PKD epithelial cells. *Biochimica et Biophysica Acta*.

[275] del Popolo G., Mencarini M., Nelli F., Lazzeri M. (2012). Controversy over the pharmacological treatments of storage symptoms in spinal cord injury patients: a literature overview. *Spinal Cord*.

[276] Matalon S., Bartoszewski R., Collawn J. F. (2015). Role of epithelial sodium channels in the regulation of lung fluid homeostasis. *American Journal of Physiology—Lung Cellular and Molecular Physiology*.

[277] Fajac I., Viel M., Sublemontier S., Hubert D., Bienvenu T. (2008). Could a defective epithelial sodium channel lead to bronchiectasis. *Respiratory Research*.

[278] Dahlberg J., Smith G., Norrving B. (2011). Genetic variants in serum and glucocortocoid regulated kinase 1, a regulator of the epithelial sodium channel, are associated with ischaemic stroke. *Journal of Hypertension*.

[279] Reddy M. M., Quinton P. M. (2003). Functional interaction of CFTR and ENaC in sweat glands. *Pflugers Archiv*.

[280] Seo I.-Y., Kim M., Lee J., Ryu S.-Y. (2008). Altered expression of sodium transporters and water channels in the submandibular gland of rats treated with nitric oxide synthesis inhibitors. *Electrolyte and Blood Pressure*.

[281] Sakamoto T., Fujii A., Saito N., Kondo H., Ohuchi A. (2016). Alteration of amiloride-sensitive salt taste nerve responses in aldosterone/NaCl-induced hypertensive rats. *Neuroscience Research*.

[282] Planes C., Caughey G. H. (2007). Regulation of the epithelial Na^+^ channel by peptidases. *Current Topics in Developmental Biology*.

[283] Shimkets R. A., Warnock D. G., Bositis C. M. (1994). Liddle's syndrome: heritable human hypertension caused by mutations in the *β* subunit of the epithelial sodium channel. *Cell*.

[284] Chilton S. N., Burton J. P., Reid G., Reid G. (2015). Inclusion of fermented foods in food guides around the world. *Nutrients*.

[285] Neta E. R. D. C., Johanningsmeier S. D., McFeeters R. F. (2007). The chemistry and physiology of sour taste—A review. *Journal of Food Science*.

[286] Lim J., Henry C. J., Haldar S. (2016). Vinegar as a functional ingredient to improve postprandial glycemic control—human intervention findings and molecular mechanisms. *Molecular Nutrition and Food Research*.

[287] Eweis D. S., Abed F., Stiban J. (2017). Carbon dioxide in carbonated beverages induces ghrelin release and increased food consumption in male rats: implications on the onset of obesity. *Obesity Research & Clinical Practice*.

[288] Hu J., Kyrou I., Tan B. K. (2016). Short-chain fatty acid acetate stimulates adipogenesis and mitochondrial biogenesis via GPR43 in brown adipocytes. *Endocrinology*.

[289] Trayhurn P. (2017). Origins and early development of the concept that brown adipose tissue thermogenesis is linked to energy balance and obesity. *Biochimie*.

[290] Bingham S. A., Gill C., Welch A. (1997). Validation of dietary assessment methods in the UK arm of EPIC using weighed records, and 24-hour urinary nitrogen and potassium and serum vitamin C and carotenoids as biomarkers. *International Journal of Epidemiology*.

[291] Pfister R., Michels G., Brägelmann J. (2014). Plasma vitamin C and risk of hospitalisation with diagnosis of atrial fibrillation in men and women in EPIC-Norfolk prospective study. *International Journal of Cardiology*.

[292] Savory L. A., Griffin S. J., Williams K. M. (2014). Changes in diet, cardiovascular risk factors and modelled cardiovascular risk following diagnosis of diabetes: 1-year results from the ADDITION-Cambridge trial cohort. *Diabetic Medicine*.

[293] Pfister R., Sharp S. J., Luben R., Wareham N. J., Khaw K.-T. (2011). Plasma vitamin C predicts incident heart failure in men and women in european prospective investigation into cancer and nutrition-norfolk prospective study. *American Heart Journal*.

[294] Lewandowski E. D., Kudej R. K., White L. T., O'Donnell J. M., Vatner S. F. (2002). Mitochondrial preference for short chain fatty acid oxidation during coronary artery constriction. *Circulation*.

[295] Bachmanov A. A., Beauchamp G. K. (2007). Taste receptor genes. *Annual Review of Nutrition*.

[296] Liang B., Soka M., Christensen A. H. (2014). Genetic variation in the two-pore domain potassium channel, TASK-1, may contribute to an atrial substrate for arrhythmogenesis. *Journal of Molecular and Cellular Cardiology*.

[297] Donner B. C., Schullenberg M., Geduldig N. (2011). Functional role of TASK-1 in the heart: studies in TASK-1-deficient mice show prolonged cardiac repolarization and reduced heart rate variability. *Basic Research in Cardiology*.

[298] Decher N., Wemhoner K., Rinne S. (2011). Knock-out of the potassium channel TASK-1 leads to a prolonged qt interval and a disturbed QRS complex. *Cellular Physiology and Biochemistry*.

[299] Rezazadeh S., Guo J., Duff H. J., Ferrier R. A., Gerull B. (2016). Reversible dilated cardiomyopathy caused by a high burden of ventricular arrhythmias in andersen-tawil syndrome. *Canadian Journal of Cardiology*.

[300] Statland J. M., Tawil R., Venance S. L. (1993). *Andersen-Tawil Syndrome*.

[301] Global. Global Market Study on Dressing Vinegar &amp; Condiments: Apple Cider Vinegar and Red Wine Vinegar Segments Projected to Gain High BPS Shares During 2016–2024. http://www.persistencemarketresearch.com/samples/13723.

[302] Panara K. B., Acharya R. (2014). Consequences of excessive use of Amlarasa (sour taste): a case-control study. *An International Quarterly Journal of Research in Ayurveda*.

[303] Cuomo R., Sarnelli G., Savarese M. F., Buyckx M. (2009). Carbonated beverages and gastrointestinal system: between myth and reality. *Nutrition, Metabolism and Cardiovascular Diseases*.

[304] Haghgou H. R., Haghgoo R., Asdollah F. M. (2016). Comparison of the microhardness of primary and permanent teeth after immersion in two types of carbonated beverages. *Journal of International Society of Preventive and Community Dentistry*.

[305] Rabie M. A., Elsaidy S., El-Badawy A.-A., Siliha H., Malcata F. X. (2011). Biogenic amine contents in selected egyptian fermented foods as determined by ion-exchange chromatography. *Journal of Food Protection*.

[306] Teener J. W. (2012). Inflammatory and toxic myopathy. *Seminars in Neurology*.

[307] Tabor D. C. (1981). Ripe and unripe: concepts of health and sickness in ayurvedic medicine. *Social Science and Medicine. Part B Medical Anthropology*.

[308] Someya A., Horie S., Yamamoto H., Murayama T. (2003). Modification of capsaicin-sensitive neurons in isolated guinea pig ileum by [6]-gingerol and lafutidine. *Journal of Pharmacological Sciences*.

[309] Shibata C., Sasaki I., Naito H., Ueno T., Matsuno S. (1999). Intragastric capsaicin stimulates motility of upper gut and proximal colon via distinct pathways in conscious dogs. *Digestive Diseases and Sciences*.

[310] Lee M.-S., Kim C.-T., Kim I.-H., Kim Y. (2011). Effects of capsaicin on lipid catabolism in 3T3-L1 adipocytes. *Phytotherapy Research*.

[311] Kang J.-H., Tsuyoshi G., Han I.-S., Kawada T., Kim Y. M., Yu R. (2010). Dietary capsaicin reduces obesity-induced insulin resistance and hepatic steatosis in obese mice fed a high-fat diet. *Obesity*.

[312] Filosa J. A., Yao X., Rath G. (2013). TRPV4 and the regulation of vascular tone. *Journal of Cardiovascular Pharmacology*.

[313] Earley S., Gonzales A. L., Garcia Z. I. (2010). A dietary agonist of transient receptor potential cation channel V3 elicits endothelium-dependent vasodilation. *Molecular Pharmacology*.

[314] Baylie R. L., Brayden J. E. (2011). TRPV channels and vascular function. *Acta Physiologica*.

[315] Fernandes E. S., Fernandes M. A., Keeble J. E. (2012). The functions of TRPA1 and TRPV1: moving away from sensory nerves. *British Journal of Pharmacology*.

[316] Bodkin J. V., Fernandes E. S. (2013). TRPV1 and SP: key elements for sepsis outcome?. *British Journal of Pharmacology*.

[317] Fernandes E. S., Liang L., Smillie S. (2012). TRPV1 deletion enhances local inflammation and accelerates the onset of systemic inflammatory response syndrome. *Journal of Immunology*.

[318] Guptill V., Cui X., Khaibullina A. (2011). Disruption of the transient receptor potential vanilloid 1 can affect survival, bacterial clearance, and cytokine gene expression during murine sepsis. *Anesthesiology*.

[319] Mendes S. J. F., Sousa F. I. A. B., Pereira D. M. S. (2016). Cinnamaldehyde modulates LPS-induced systemic inflammatory response syndrome through TRPA1-dependent and independent mechanisms. *International Immunopharmacology*.

[320] Chen Y., Fang Q., Wang Z. (2016). Transient receptor potential vanilloid 4 ion channel functions as a pruriceptor in epidermal keratinocytes to evoke histaminergic itch. *Journal of Biological Chemistry*.

[321] Akiyama T., Ivanov M., Nagamine M. (2016). Involvement of TRPV4 in serotonin-evoked scratching. *Journal of Investigative Dermatology*.

[322] Andersen H., Melholt C., Hilborg et al. S. (2017). Antipruritic effect of cold-induced and transient receptor potential-agonist-induced counter-irritation on histaminergic itch in humans. *Acta Dermato Venereologica*.

[323] Yu G., Yang N., Li F. (2016). Enhanced itch elicited by capsaicin in a chronic itch model. *Molecular Pain*.

[324] de La Iglesia R., Milagro F. I., Campion J., Boque N., Martínez J. A. (2010). Healthy properties of proanthocyanidins. *BioFactors*.

[325] Lu B., Wang T., Li Z. (2016). Healing of skin wounds with a chitosan-gelatin sponge loaded with tannins and platelet-rich plasma. *International Journal of Biological Macromolecules*.

[326] Chung K.-T., Wong T. Y., Wei C.-I., Huang Y.-W., Lin Y. (1998). Tannins and human health: a review. *Critical Reviews in Food Science and Nutrition*.

[327] Lim S. W. (2012). Aluminum potassium sulfate and tannic acid injection for hemorrhoids. *Journal of the Korean Society of Coloproctology*.

[328] Kim K., Lee H., Hong S. (2016). TAPE: a biodegradable hemostatic glue inspired by a ubiquitous compound in plants for surgical application. *Journal of Visualized Experiments*.

[329] Bas J. M. D., Ricketts M.-L., Vaque M. (2009). Dietary procyanidins enhance transcriptional activity of bile acid-activated FXR in vitro and reduce triglyceridemia in vivo in a FXR-dependent manner. *Molecular Nutrition and Food Research*.

[330] Jadoun A., Dwivedi R. (2013). Effect of selected samana and vicitra pratyayarabdha dravya w.s.r. to vipaka. *An International Quarterly Journal of Research in Ayurveda*.

[331] Gilca M., Dragos D., Irina S. (2016). *Medicinal plants and biologically active phytochemicals. Iasi*.

[332] Garcia-Hernandez K. Y., Vibrans H., Rivas-Guevara M., Aguilar-Contreras A. (2015). This plant treats that illness? the hot-cold system and therapeutic procedures mediate medicinal plant use in San Miguel Tulancingo, Oaxaca, Mexico. *Journal of Ethnopharmacology*.

[333] Ankli A., Sticher O., Heinrich M. (1999). Yucatec maya medicinal plants versus nonmedicinal plants: indigenous characterization and selection. *Human Ecology*.

[334] Parvinroo S., Zahediasl S., Sabetkasaei M., Kamalinejad M., Naghibi F. (2014). The effects of selected hot and cold temperament herbs based on Iranian traditional medicine on some metabolic parameters in normal rats. *Iranian Journal of Pharmaceutical Research*.

[335] Gonzales de La Cruz M., Baldeon Malpartida S., Beltran Santiago H., Jullian V., Bourdy G. (2014). Hot and cold: medicinal plant uses in Quechua speaking communities in the high Andes (Callejon de Huaylas, Ancash, Peru). *Journal of Ethnopharmacology*.

[336] Tedlock B. (1987). An interpretive solution to the problem of humoral medicine in Latin America. *Social Science and Medicine*.

[337] Ardekani M. R. S., Rahimi R., Javadi B., Abdi L., Khanavi M. (2011). Relationship between temperaments of medicinal plants and their major chemical compounds. *Journal of Traditional Chinese Medicine*.

[338] Fu X.-J., Wang Z.-G., Qu Y., Wang P., Zhou Y., Yu H.-Y. (2013). Study on the networks of "nature-family-component" of Chinese medicinal herbs based on association rules mining. *Chinese Journal of Integrative Medicine*.

[339] Chao D.-P., Chen J.-J., Huang S.-Y., Tyan C.-C., Hsieh C.-L., Sheen L.-Y. (2011). Effects of hot and cold foods on signals of heart rate variability and nail fold microcirculation of healthy young humans: a pilot study. *Chinese Journal of Physiology*.

[340] Chao D.-P., Tyan C.-C., Chen J.-J., Hsieh C.-L., Sheen L.-Y. (2011). Effect of hot-attribute aged ginger tea on chinese medical pulse condition of healthy young humans. *Journal of Traditional and Complementary Medicine*.

[341] Parvinroo S., Naghibi F., Zahediasl S., Kamalinejad M., Sabetkasaei M. (2014). The effects of seeds with hot and cold temperaments on serum thyroid hormones, corticosterone and urine vanillylmandelic acid concentrations of healthy rats. *Journal of Ethnopharmacology*.

[342] Hlang L.-P., Zhu M.-F., Yu R.-Y., Du J.-Q., Liu H.-N. (2014). Study on discrimination mode of cold and hot properties of traditional chinese medicines based on biological effects. *Zhongguo Zhong Yao Za Zhi*.

[343] Dhaka A., Earley T. J., Watson J., Patapoutian A. (2008). Visualizing cold spots: TRPM8-expressing sensory neurons and their projections. *Journal of Neuroscience*.

[344] Fujiyama R., Toda K. (2016). Functional effects of cold stimulation on taste perception in humans. *Odontology*.

[345] Medler K. F. (2011). Multiple roles for TRPs in the taste system: not your typical TRPs. *Advances in Experimental Medicine and Biology*.

[346] Lyall V., Heck G. L., Vinnikova A. K. (2004). The mammalian amiloride-insensitive non-specific salt taste receptor is a vanilloid receptor-1 variant. *Journal of Physiology*.

[347] Levine J. D., Alessandri-Haber N. (2007). TRP channels: targets for the relief of pain. *Biochimica et Biophysica Acta: Molecular Basis of Disease*.

[348] Zhao R., Tsang S. Y. (2016). Versatile roles of intracellularly located TRPV1 channel. *Journal of Cellular Physiology*.

[349] Viana F. (2011). Chemosensory properties of the trigeminal system. *ACS Chemical Neuroscience*.

[350] Bufe B., Breslin P. A. S., Kuhn C. (2005). The molecular basis of individual differences in phenylthiocarbamide and propylthiouracil bitterness perception. *Current Biology*.

[351] Prutkin J., Duffy V. B., Etter L. (2000). Genetic variation and inferences about perceived taste intensity in mice and men. *Physiology and Behavior*.

[352] Bartoshuk L. M. (2000). Comparing sensory experiences across individuals: recent psychophysical advances illuminate genetic variation in taste perception. *Chemical Senses*.

[353] Duffy V. B., Peterson J. M., Bartoshuk L. M. (2004). Associations between taste genetics, oral sensation and alcohol intake. *Physiology and Behavior*.

[354] World Health Organisation (2011). *Quality Control Methods for Herbal Materials*.

[355] Reinberger S. (2006). Bitter could be better. *Scientific American Mind*.

[356] Leonti M. (2011). The future is written: impact of scripts on the cognition, selection, knowledge and transmission of medicinal plant use and its implications for ethnobotany and ethnopharmacology. *Journal of Ethnopharmacology*.

[357] Martin L. J., Sollars S. I. (2017). Contributory role of sex differences in the variations of gustatory function. *Journal of Neuroscience Research*.

[358] Ley J. P. (2008). Masking bitter taste by molecules. *Chemosensory Perception*.

[359] Imming P., Sinning C., Meyer A. (2006). Drugs, their targets and the nature and number of drug targets. *Nature Reviews Drug Discovery*.

[360] Roland W. S. U., Gouka R. J., Gruppen H. (2014). 6-Methoxyflavanones as bitter taste receptor blockers for hTAS2R39. *PLoS ONE*.

[361] Cocco N., Glendinning J. I. (2012). Not all sugars are created equal: some mask aversive tastes better than others in an herbivorous insect. *Journal of Experimental Biology*.

[362] Behrens M., Born S., Redel U. (2012). Immunohistochemical detection of TAS2R38 protein in human taste cells. *PLoS ONE*.

[363] Voigt A., Hübner S., Lossow K., Hermans-Borgmeyer I., Boehm U., Meyerhof W. (2012). Genetic labeling of Tas1r1 and Tas2r131 taste receptor cells in mice. *Chemical Senses*.

[364] Reyes-Garcia V. (2010). The relevance of traditional knowledge systems for ethnopharmacological research: theoretical and methodological contributions. *Journal of Ethnobiology and Ethnomedicine*.

